# Investigating the Role of Communication for Information Seekers’ Trust-Related Evaluations of Health Videos on the Web: Content Analysis, Survey Data, and Experiment

**DOI:** 10.2196/10282

**Published:** 2018-12-21

**Authors:** Maria Zimmermann, Regina Jucks

**Affiliations:** 1 Department of Psychology and Sport Science Institute for Psychology in Education Münster Germany

**Keywords:** trust, health communication, social media, information-seeking behavior, language

## Abstract

**Background:**

According to the language expectancy theory and the communication accommodation theory, health information seekers’ trust evaluations of Web-based videos are determined by interplays between content and seekers’ expectations on vloggers’ appropriate language use in specific contexts of Web-based communication.

**Objectives:**

Two investigations focused on differences both between vloggers’ language styles and between users’ general trust in specific Web-based platforms to investigate how the context of Web-based communication can be characterized (research question, RQ1). Thereafter, we investigated whether information uncertainty, vloggers’ language style, and context of Web-based communication affect seekers’ trust evaluations of videos (RQ2).

**Methods:**

With a content analysis of 36 health videos from YouTube and Vimeo, we examined the extent of trust-related linguistic characteristics (ie, first-person and second-person pronouns). Additionally, we surveyed participants (n=151) on their trust in YouTube and Moodle (academic Web-based platform; RQ1). In an experiment, further participants (n=124) watched a video about nutrition myths and were asked to evaluate the information credibility, vloggers’ trustworthiness, and accommodation of language by vloggers (RQ2). Following a 3 × 2 × 2 mixed design, vloggers’ explanations contained unambiguous (confirming or disconfirming) or ambiguous (neither confirming nor disconfirming) evidence on the myths (within factor). Furthermore, vloggers used YouTube-typical language (many first-person pronouns) or formal language (no first-person pronouns), and videos were presented on YouTube or Moodle (between factors).

**Results:**

The content analysis revealed that videos on YouTube contained more first-person pronouns than on Vimeo (*F*_1,35_=4.64; *P*=.04; η_*p*_^2^=0.12), but no more second-person pronouns (*F*_1,35_=1.23; *P*=.23). Furthermore, when asked about their trust in YouTube or Moodle, participants trusted YouTube more than Moodle (*t*_150_≤−9.63; all *P* ≤.001). In the experiment, participants evaluated information to be more credible when information contained unambiguous rather than ambiguous evidence (*F*_2,116_=9.109; *P*<.001; η_*p*_^2^=0.14). Unexpectedly, information credibility did not depend on vloggers’ language style or the video platform (*F*_1,117_≤2.40; *P* ≥.06). Likewise, video’s platform did not affect participants’ evaluations of vloggers’trustworthiness (*F*_1,117_<0.18; *P*>.34). However, participants judged vloggers who used a YouTube-typical language as being more benevolent, and their language use as being more appropriate in both video platforms (*F*_1,117_≥3.41; *P* ≤.03; η_*p*_^2^≥0.028). Moreover, participants rated the YouTube-typical (vs formal) language as more appropriate for Moodle, but they did not rate one or the other language style as more appropriate for YouTube (*F*_1,117_=5.40; *P*=.01; η_*p*_^2^=0.04).

**Conclusions:**

This study shows that among specific Web-based contexts, users’ typical language use can differ, as can their trust-related evaluations. In addition, health information seekers seem to be affected by providers’ language styles in ways that depend on the Web-based communication context. Accordingly, further investigations that would identify concrete interplays between language style and communication context might help providers to understand whether additional information would help or hurt seekers’ ability to accurately evaluate information.

## Introduction

### Trust in Web-Based Nutrition Information

At least because of Popeye whose arms triple when he eats spinach and gains enormous power, the folk wisdom about spinach and its immense amount of iron has become anchored in the minds of the people. Even the information about the malpositioned decimal point that caused the falsely perceived iron content of spinach—and the followed scientific discussion about citation errors that have spread this false assumption—have not changed much [1].

Whenever people have questions about nutrition, the easiest way they can get information is on the internet. Using the internet has become a widespread way to inform people about nutrition [2,3], and how people process and evaluate Web-based health information affects their medical decisions as well as their health [4-7]. Although it can be challenging for people to get accurate information on the internet [2,4,8], the internet concurrently also offers the advantages of interactivity, anonymity, and a low threshold for getting information.

Regarding getting information from Web-based videos, people reported that they watch Web-based videos not only to gain knowledge [9] but also to support their individual learning [10,11]. However, viewing videos from video platforms on the Web may put (lay)people at risk of getting inaccurate, incomplete, or inadequate information not only because the content of Web-based videos is rarely reviewed and, therefore, the quality varies vastly [12,13] but also because information seekers have difficulty evaluating whether the obtained information is true [7]. In this sense, determining the objective accuracy of any health information (video or otherwise) is challenging as even medical evidence rarely attains absolute certainty and can always be proved wrong by supplemental evidence [7,14].

Thus, information seekers’ risk of encountering inaccurate information on the Web comes from both the proportion of information that is likely inaccurate and the inability of a (lay)person to filter inaccurate information [8]. Consequently, information seekers need to rely on qualified information providers, as their own expertise about the topics of interest is likely incomplete, and their resources for evaluating the content according to academic criteria are limited (eg, evaluating whether the evidence given to support a claim is based on plausible scientific methods [4,15]). Instead, they need to identify criteria that indicate whom to rely on [7]. Therefore, in addition to or instead of understanding the academic criteria that can be used to assess the actual quality of information, it is also critically important to understand what kind of criteria information seekers use to determine that they can trust someone to provide adequate information [7,8,16,17].

Although various phenomena related to trust are mirrored in diverse research fields—leading also to many definitions of trust [18-21]—we consider the common principles of trust when examining the relationship between information seekers and providers. As such, relationships between information seekers (trustors) and providers (trustees or trust objects) [22-25] entail an actual or perceived imbalance in each party’s extent of knowledge. In turn, trusting the information provider requires that the information seeker willingly accepts that he or she can never really know if the information is adequate or whether the provider indeed has more knowledge (uncertainty of trustors [21-23,25,26]). Thus, health information seekers’ risk being misinformed if the information provider offers inaccurate, incomplete, or inadequate information. In this sense, several characteristics of trust objects (such as the provider’s trustworthiness or attractiveness) are considered antecedents of trust. Moreover, (dis)trust is considered to be either an outcome from, or a formative, or a conditional aspect of a trusting or a distrusting relationship [23,25,27].

So far, the research on seekers’ evaluation of Web-based information has shown that several factors, such as the information source, the content of the information, and the type of media, are important when people evaluate the credibility of Web-based information in general [28-32]. Regarding health information specifically, the kind of criteria that people use to judge the credibility of Web-based health information is often related to the characteristics of the Web-based information and the provider of the Web-based information [16,22]. Although people’s judgments of a provider’s trustworthiness correspond to judgments about the credibility of information and vice versa [33], single factors, such as the provider’s language and the content of information, seem to influence information seekers’ evaluations of the information and the provider in different ways [34,35]. Therefore, in the rest of the study, we consider that people’s willingness to rely on both the characteristics of the provided information and those of the information provider are concepts (hereinafter called the *credibility of information* and the *trustworthiness of providers*) that, although they depend on one another, can be influenced differently by individual factors.

The main aim of this study was to extend the research on how health information seekers evaluate information and information providers. We consider the relationship between health information seekers and providers to be mainly determined communicative patterns. Thus, in the following sections, we outline how the information providers’ language style and the context of the Web-based communication can be viewed as factors that information seekers analyze to determine whom and which information to rely on.

### The Communication Between Health Information Seekers and Providers

According to language expectancy theory (LET) [36] and communication accommodation theory (CAT) [37], gaining knowledge when seeking health information on the Web is not only determined by the content of the information (ie, what is said) but also by who is communicating (ie, who is saying it), their manner of communication (ie, how are they saying it), and the context of communication (ie, *where* takes the communication place). In particular, LET predicts people’s attitude change (eg, imparting of knowledge) on any violations of their language expectancies that represent people’s expectations on interlocutors’ appropriate language use in a given context. Accordingly, information seekers should evaluate providers positively if providers use language styles that fit with cultural values and situational norms (ie, no violations). Similarly, information seekers should evaluate providers positively if providers use language styles more favorably than expected in a situation (ie, positive violation, eg, a doctor uses unexpectedly easy everyday terms as the doctor was used to use difficult medical jargon at previous appointments). Contrary, they should evaluate providers negatively if providers use language that conflicts with cultural values and situational norms (negative violations). Similar to what LET predicts, the CAT also predicts that information providers should be evaluated more positively if they use appropriate (ie, accommodative) language in a specific context. According to CAT, the interlocutor or audience is an important aspect that determines the contextual norms of communication.

In sum, both theories on interpersonal communication take into account the context of communication. In this sense, LET and CAT consider that the rules and norms embedded in the context of communication influence how people communicate, how people evaluate their interlocutors, and how people evaluate the appropriateness of their interlocutors’ language use given a certain context; hence, these factors influence the communicative success.

### Health Information Providers’ Language Styles

On the Web, the language style used by the information provider offers especially salient information about the provider [15,16,38,39] and, therefore, is likely to influence an information seeker’s perceived trustworthiness of the provider [5,15,38-43]. In this sense, an information seeker might perceive a specific language style used by the provider and then use it to judge some characteristics of the provider such as their competence or benevolence. In general, one’s language style can be characterized by numerous linguistic aspects (eg, the use of technical terms, self-references, or hedges), and a health information provider’s language style might impact a seeker’s judgments about their credibility and trustworthiness [34,35,40,41].

Information providers’ use of personal references (ie, referring to oneself or someone else) is closely associated with how people perceive providers’ expertise [40,41] and how much people learn from them [44], and hence, such references are of special interest for seeking health information. Linguistically, as self-references (eg, first-person pronouns) are related to the extent of a speaker’s self-disclosure, they might offer information about providers’ trustworthiness in 2 ways: on the one hand, self-disclosure is considered important for establishing trust in Web-based communication, as it signals a willingness to open up and, hence, might promote the reciprocal exchange of information [38]; accordingly, high number of self-references can lead to higher credibility judgments [45]. On the other hand, it seems crucial that people share just the right amount of personal information, as sharing too much personal information and using too many first-person pronouns can also be perceived as unprofessional [41]; accordingly, high number of self-references are also found to negatively influence the trustworthiness of information providers on the Web [40,46]. Overall, the evidence on how self-disclosure affects trust-related evaluations is sparse and conflicting. Similarly, research has revealed conflicting evidence on how providers’ use of technical language (also associated with providers’ trustworthiness [41]) affects credibility judgments, as using high amounts of technical language has led to both higher [41,47] and lower credibility judgments [48,49]. These conflicting findings on the individual impact of providers’ language styles on trust-related evaluations might strengthen the assumptions of LET and CAT, which state that people’s evaluations of providers are not only influenced by the language style itself but by a complex interaction among the content, the interlocutors, the provider’s language use, and the context of communication.

### The Context of Web-Based Communication

*Context* is considered crucial in several approaches on people’s evaluations of Web-based information [5,32,50-56]. Although context is always assumed to influence people’s evaluations, what constitutes this context has been defined, however, with different levels of concreteness and using different aspects [56]. For instance, context has previously been described in terms of how long people spend searching for Web-based information [53,57], the characteristics of information seekers (such as their self-disclosure, expectations, or experiences) [52,58], the interaction of several aspects [50,56], or differences between seeking information on the Web or in *naturalistic* (ie, offline) settings [59].

Although context has often been assumed to influence people’s evaluations, it has been, so far, rarely investigated in research about credibility on the Web, which may be because of the challenges in defining and conceptualizing context for Web-based as well as offline information seeking [33,56]. However, a review of the theoretical frameworks used to assess Web-based information emphasizes that to operationalize credibility, considerations, and conceptualizations of different Web-based contexts need to be outlined, as we must investigate these contexts to understand people’s credibility judgments better [33]. Furthermore, Web-based health information seekers risk misjudging even accurate information if they do not recognize the context in which the information is supposed to be interpreted (also described as *context deficit* [5,60]).

By combining the concept of context in LET and CAT with approaches that have been used to study the impact of online media and its affordances (relating to its features) on people’s evaluation of Web-based information [32,61], we consider the context of Web-based communication to cover the norms and rules about users’ typical use of online media as well as users’ expectations of typical media use. Expectations of typical media use relate to the affordances that online media offer (such as navigability or recordability), where *affordances* refer not only to the simple features but also to the dynamic relationships among those features, the cues (eg, *button* for record) that features offer users, and how users use those cues [61]. Importantly, these affordances should be considered as part of the Web-based context, as research has considered several affordances of online media that might affect trust-related evaluations of Web-based information and information providers [30,32,35,62].

By considering the concept of online media affordances, we derived the following definition for the Web-based communication context.

The Web-based communication context is determined by certain norms. These norms emerge from and shape continuous dynamic relationships among (1) affordances of online media, (2) cues accompanying these affordances, and (3) how users use these cues [63].

This definition complements the concept of affordances by adding the concept of norms, which includes users’ typical use and users’ expectations on typical use. Norms or rules should constitute and emerge from users’ (typical) use of online media—irrespective of explicitly named rules of the media. For instance, if specific affordances of online media create norms on how to communicate typically, these norms should also entail users’ expectations about an appropriate way to communicate in this media [64]. In this sense, the community of online media (ie, users) is crucial for establishing norms and, hence, is an important aspect of the context [37]. Thus, the definition of contexts closely refers to research on norms of online communities: for instance, group member’s awareness of norms that were caused by online communities’ characteristics (ie, size and extent of use, lack of nonverbal cues, anonymity, warranting, communication record, and community type [65]), norms of online communities that are caused by sociotechnical interactions with characteristics of the Web-based environment [66], or sharing norms as interactions within virtual communities [67]. In this sense, it is likely that online communities may also evoke norms for community-specific language styles [64].

### Previous Research on the Impact of Contexts of Web-Based Communication

With respect to rather typical language styles for specific contexts of Web-based communication, research has identified differences in linguistics characteristics between Facebook, Twitter, and YouTube, where the affordances of the media platform cause certain differences such as the length of the texts: Facebook posts can contain unlimited numbers of characters, whereas Twitter posts (tweets) are limited to 140 characters [68]. Similarly, YouTube’s affordances of high anonymity and heterogeneity among users may cause more foul language on YouTube than on Facebook [69].

With respect to how the context of Web-based communication affects trust-related evaluations, previous studies have shown that users are affected by different types of online media itself (ie., websites, blogs, bulletin boards, and internet) [70] as well as by different affordances and cues related to a type of online media [71,72]. With respect to the community as an important aspect of Web-based contexts, information seekers evaluated medical experts as being more trustworthy when they provided information in online forums for medical professionals rather than in online forums for lay persons; hence, seekers evaluated the trustworthiness of medical experts differently depending on the specific online community [34].

Moreover, research also indicates that the appropriateness of a health information provider’s language style is crucial not only when health information is sought offline [73,74] but also when it is sought on the Web [34,75-79]. In this sense, when providers use language that is accommodative toward the (online) audience, this is considered appropriate, as it takes into account the audience’s communication preferences and, in turn, enhances their satisfaction with the communication [37,74].

As users of online media are considered to show and expect the norms of a particular (Web-based) context of communication [37,65], it is interesting that some of the research done so far, although there are not many studies, have identified differences in users’ language styles for different online media [68,69]. Furthermore, 1 study also found that using a particular language style (ie, the number of self-references in messages) led users to evaluate a provider’s trustworthiness differently depending on whether the language style was used on Facebook or Twitter [62]. Thus, participants’ evaluations were seemingly impacted by the provider’s language style as well as by the context of the Web-based communication. Overall, research indicates that users of different Web-based platforms communicate in different ways, and they evaluate information and providers’ language styles differently depending on the context of Web-based communication.

### Rationale

Seeking health information on the Web is a common way to obtain knowledge about health-related information and can influence personal health decisions [6]. In this sense, health information seekers need to use criteria that indicate whom they can trust to provide accurate, complete, and adequate information [7]. As such, it is important to identify these criteria and investigate their impact on trust-related evaluations. As seeking health information on the Web involves communication between information seekers and providers, trust-related evaluations depend not only on the content of the information but also on how seekers evaluate the providers and their manner of communicating and also on the context in which the communication takes place. Moreover, information seekers’ evaluations also are shaped by their expectations about appropriate language use, given a specific context [36,37]. According to LET and CAT, health information providers’ language styles and the context of Web-based communication should, therefore, impact health information seekers’ evaluations of providers and the information in 2 ways, individually and reciprocally. However, research considering both aspects is sparse.

As it is difficult to conceptualize the context of Web-based communication, we aimed to identify differences between specific Web-based contexts that are associated with users’ typical use [36,37,61], as this would not only extend previous findings on Web-based context-specific norms but it would also help to characterize specific contexts more concretely. For this purpose, we investigated differences in users’ language styles (first investigation) and users’ trust evaluations (second investigation) regarding specific contexts of Web-based communication that are relevant when watching Web-based health videos. As such, the Web-based communication context is defined by norms and rules about the online media affordances, cues accompanying affordances, and how users use these cues. Accordingly, diverse Web-based platforms may adequately represent and operationalize different contexts of Web-based communication, as users’ use of Web-based platforms and these platforms’ affordances should constitute the norms corresponding to users’ typical use of the Web-based platforms.

Research question (RQ) 1: Are there differences in users’ language styles and users’ trust-related evaluations regarding specific contexts of Web-based communication?

Building on the findings of this study’s first and second investigations, which address RQ1, this study’s third investigation examined experimentally whether health information seekers’ trust-related evaluations of a Web-based video about nutrition myths were affected by the content of information, the language style of the information provider, and the context of the Web-based communication.

RQ2: Here, we consider an interaction between the language style of health information providers and the context of Web-based communication: are health information seekers’ willing to rely on information providers and the information they provide impacted by the information itself, the information provider’s language style, the appropriateness of this language styles given the specific context of Web-based communication, and the context of Web-based communication?

The aim of this study’s first 2 investigations was to gain empirical insights into certain contexts of Web-based communication. We, therefore, explored whether people communicate and trust differently depending on the Web-based context—namely, the Web-based platform. Accordingly, we first investigated whether speakers of Web-based health videos on Vimeo or YouTube used different linguistic styles. As we further aimed to extend the research on how Web-based platforms individually impact users’ trust evaluations [70], we, in a second study, investigated whether users differ in their willingness to trust YouTube and Moodle (a platform for universities to share learning material). The empirical findings from both investigations allowed us to combine the theoretical assumptions about the context of Web-based communication derived above with actual findings from specific Web-based platforms.

### Background for Investigation 1: Vloggers’ Language Styles on YouTube and Vimeo

Although YouTube is arguably the most popular social media platform to watch, share, and comment on Web-based videos (eg, based on monthly unique viewers [80]), we aimed to compare YouTube with a Web-based platform that is similar in its purpose and is also popular. Thus, we selected Vimeo, belonging to InterActiveCorp, which is also a social media platform to watch, share, and comment on Web-based videos; it has about 60 million registered users [81] and is used by about 240 million unique users per month. As such, Vimeo has significantly fewer users than YouTube, which is used by over a billion people [82]. Moreover, every day 5 billion videos are watched on YouTube, and every minute 300 hours of video material is added [83]. Together, YouTube and Vimeo were accessed most frequently by internet users to watch videos in the United States [84], with Vimeo, after YouTube, reaching the second highest extent of internet users in the United Kingdom [85]. Furthermore, YouTube and Vimeo differ with respect to their financing. Although the free version of YouTube—which is probably used by users most often—is mainly financed by advertising displayed before and during the videos, the ad-free platform Vimeo offers both free memberships and *pro* memberships, and the whole platform is financed exclusively through these pro memberships [81]. As such, Vimeo also offers services that can increase the quality of videos, and it further curates films to provide high-quality content.

Therefore, YouTube and Vimeo can be considered examples of Web-based platforms whose differences might lead to community-specific norms, which may result in community-specific language styles [36,37,64], but that are still comparable in terms of most users’ purposes for use. So far, however, only a few studies have identified linguistic differences between Web-based platforms [68,69]. In 1 study, a content analysis of contributions on Web-based platforms revealed that nonprofit advocacy groups use different propaganda characteristics in their messages on Facebook, Twitter, and YouTube [86]. When posting on YouTube compared with other platforms, these organizations used more authority figures; conversely, when posting on Twitter compared with Facebook or YouTube, they more often reduced complex issues. Similarly, users seemed to use more foul language on YouTube compared with other platforms like Facebook, which might because YouTube has heterogeneous users and is highly anonymous [69].

We aimed to extend these findings by investigating whether information providers in Web-based health videos use different number of personal references on Vimeo or YouTube. We focused on first- and second-person pronouns as they represent the use of personalization in language styles in Web-based videos [13,87] and are crucial for trust-related evaluations [38,40,41].

### Background for Investigation 2: Users’ Trust in YouTube and Moodle

As we aimed to investigate differences in users’ trust-related evaluations in different Web-based contexts of communication, we investigated whether users’ trust in Web-based platforms differs. In this sense, it is worthwhile to determine if users do trust in Web-based platforms to provide Web-based services because trust in these trust objects (services) might also affect trust in associated trust objects (the information on the platforms). Accordingly, any differences between users’ trust in these platforms additionally allow one to derive hypotheses about whether such platforms have individual effects on information seekers’ evaluations of the information provider and the information (this study’s third investigation). In this sense, differences between how users evaluate the same information (regarding whether they trust it) that occurs on different Web-based platforms [70] might be caused by users’ general trust in those platforms [30,88].

We decided to investigate users’ trust in YouTube versus in a Web-based platform that obviously differs in its operators but still offers users the opportunity to watch Web-based videos. YouTube and Moodle represent good platforms to compare, as Moodle is a platform provider for educational institutions, and its main purpose is to share learning materials such as book chapters, course curricula, and videos; conversely, YouTube is the most popular social media platform to watch, share, and comment on Web-based videos.

Research on trust in Web-based platforms [89-92] considers trust similarly to how we have described it above, where research aims to both investigate trust as a formative aspect leading to trust-related actions regarding the trust object (such as using a Web-based platform [89,90]) as well as to investigate characteristics of Web-based platforms as antecedents of trust (such as trustworthiness or usability [91,92]). To investigate whether there are differences between both platforms in terms of people’s trust in the platform, we assessed people’s perception of each platform’s characteristics and people’s self-reported willingness to interact with the platform as well as people’s self-reported familiarity with the platform when using for a certain purpose [86].

### Background for Investigation 3: The Impacts of the Uncertainty of Information, Information Provider’s Language Style, and the Context of Web-Based Communication on Health Information Seekers’ Trust-Related Evaluations for a Web-Based Video About Nutrition Myths

According to LET and CAT, whether health information seekers rely on information providers and their provided information depends not only on the content of information but also on the providers’ language styles, the contexts of Web-based communication, as well as on seekers’ expectancies about appropriate language styles, given specific Web-based contexts (ie, the reciprocal impact of language styles and contexts of Web-based communication). In this sense, research indicates that information seekers make use of not only single factors related to information providers, the information itself, or the media through which information is transferred when evaluating Web-based information [29,33,34,46]. Thus, this study’s third investigation intended to investigate how information seekers are affected by the integrative consideration of single factors at these levels. Hence, building on the findings of this study’s first 2 investigations, the following experiment examined how the uncertainty of information, the language style of the provider, and the context of Web-based communication affect individually and reciprocally seekers’ evaluations of information credibility and the provider’s trustworthiness when watching a Web-based video about nutrition myths (RQ2). Therefore, a 3 × 2 × 2 mixed-design Web-based experiment was conducted with the factors being uncertainty of information (uncertain, confirming the myth, and not confirming the myth), language style of the vlogger (YouTube-typical vs formal language style), and the context of Web-based communication (Moodle vs YouTube).

The Web-based platforms YouTube and Moodle were chosen as contexts of Web-based communication because they contain features that should determine differences between users’ typical use of both platforms and should, therefore, impact health information seekers’ trust-related evaluations. Moodle is a platform provider for educational institutions, and the main purpose of the use is to share learning materials, such as book chapters, course curricula, and videos. Accordingly, videos on Moodle are most often uploaded by academics who are providing learning material to their students. Conversely, videos on YouTube are not necessarily uploaded by academics because every user is allowed to upload content for several purposes. Hence, on Moodle, the homogenous community is made up of rather *educationally close* members compared with the heterogenous community on YouTube. As the community (ie, users) of both Web-based platforms differs, it is likely that these platforms represent contexts of Web-based communication with different norms [36,37,65]. Accordingly, the platforms should impact how users communicate on these Web-based platforms and how they evaluate the appropriateness of information providers’ language use [64]. In addition, users on both platforms can watch Web-based videos, but users seem to trust in these platforms differently. In particular, people seem to be more familiar and more willingness to transact with YouTube compared with Moodle, whereas they do not ascribe higher ability, benevolence, nor integrity to YouTube compared with Moodle (this study’s second investigation).

In the following, we summarize the theoretical and empirical approaches for each factor and the interplay among a vlogger’s language style and the context of Web-based communication to derive hypotheses in terms of factors’ individual and reciprocal impacts.

#### Uncertainty of Health Information on the Web

Research on the credibility of Web-based information indicates that the content of information affects people’s evaluation [29,33,35]. Thereby, the uncertainty of information has been highlighted in several studies because often scientific information entails preliminary findings—which are further often discussed controversially, especially if findings’ origins and implications are attributed to conflicting scientific trends [93]. On the Web, seeking health information can bring to light the uncertainty of information, as people not only become aware of conflicting opinions about symptoms’ origins, diagnosis validity, and optimal treatments but they can also be confronted with numerous conflicting opinions [94,95]. By focusing on ambiguity as an antecedent of uncertainty, the ambiguous representation of an issue has been shown to make people see evidence as risky and avoid making decisions, and these outcomes appear to depend on the origin of the ambiguity—for instance, whether the information is either conflicting or incomplete [96].

Accordingly, people’s confusion has also been investigated for conflictual information in Web-based newspapers [97]. In that work, studies with more conflictual information about deep brain stimulation was associated with people expressing higher levels of uncertainty and commenting more negatively on these studies. Similarly, people judged an original message about the risk of red meat to be more credible if they received another message about its risks (a consistent message) compared with a message about its benefits (a conflicting message); interestingly, these conflicting and consistent messages did not influence the perceived trustworthiness of the information provider [98]. In addition, conflicting rather than consistent information about the risky use of a drug to treat high cholesterol made participants less likely to recommend the medication to a fictitious friend [95]. However, in a study where people received analogous information about both the risks and benefits of either an unfamiliar or a familiar food supplement (ie, conflicting information), people reflected more about these risks and benefits if they generally believe knowledge to be dynamic and complex and if they were given information about the less familiar (CQ10) supplement [99]. This, in turn, indicates that people’s epistemic beliefs and their familiarity with the topic influence how they process ambiguous information. In general, people seem to heuristically use rules of thumb when receiving conflicting instead of consistent information, and therefore, they seemingly use different strategies to decide about their health depending on whether they are processing conflicting or consistent information [100].

Although these findings predominantly indicate that people seem to perceive consistent information to be more credible than conflicting information, people’s judgments in this context are, for instance, also influenced by the following factors: the order of presented information, people’s prior epistemic beliefs, and people’s attitude toward the topic [101]; the complexity of information and the number of sources that provide the information [102]; and the overall trustworthiness of the information provider [103]. Hence, again, instead of considering only the uncertainty of information, it is more realistic to also consider characteristics regarding the provider of information.

As nutrition myths are widespread and collective conceptions about nutrition arise because they are reiterated over and over, people’s familiarity with such (mis)conceptions causes them to believe that the (mis)information is correct—irrespective of the scientific origin of the information [1,104,105]. After considering the summarized evidence for how people evaluate the uncertainty of Web-based information, we have derived the following presumptions and accompanying hypotheses for how people evaluate Web-based videos about nutrition myths:

H1: When discussing a popular nutrition myth, a vlogger’s scientific explanations that confirm, disconfirm, or neither confirm nor disconfirm the myth are expected to impact how people judge the credibility of information. People should evaluate a video’s information that is consistent with the nutrition myth (confirming the myth) as being more credible compared with a video that gives conflicting information (disconfirming the myth) or that gives information that neither confirms nor disconfirms the nutrition myth. In addition, people should evaluate a video’s information to be more credible when scientific explanations neither confirm nor disconfirm the myth compared with those explanations that disconfirm the nutrition myth.

#### Language Style of Vloggers

According to LET and CAT, the success of seeking health information on the Web is determined by how health information seekers evaluate the providers’ language style. Especially on the Web, the language style of an information provider is considered an outstanding cue to assess characteristics about the provider [15,16,38,39]. Indeed, research, so far, indicates that the provider’s language style is a cue that impacts whether people perceive the information to be credible and the information provider to be trustworthy [34,35,40,41].

In this context, linguistic aspects of personal references (eg, first-person and second-person pronouns) are of special interest for seeking health information, as providers’ use of personal references is closely associated with providers’ expertise [35,40,41]. According to the findings in this study’s first study, a typical characteristic of health videos on YouTube is the usage of first-person pronouns. Similarly, so-called conversational language is achieved by linguistic aspects of self-disclosure [106]: That is, changing third-person into first-person pronouns makes it seem as if the author (or vlogger) is talking to you and is expressing a personal experience. As a YouTube-typical language style involves self-disclosure by the communicator, it might be an important mechanism for establishing trust in Web-based communication [38,41]. However, it is unclear to what extent sharing personal information might promote a trustful relationship: it could be that sharing personal information entails the risk of reducing the perceived expertise of the provider, as experts are expected to conduct themselves in an objective and unbiased manner. Accordingly, fewer first-person pronouns in Web-based medical advice predicted a higher perceived expertise of advice givers and helped people to determine that the advice givers were experts rather than laypersons [41]. Similarly, mental health information in messages on Facebook and Twitter that included personal testimonials (compared with those without personal testimonials) led people to think more critically about the provider [46]. In addition, another study showed that high self-disclosure in Web-based advice negatively impacted the perceived benevolence of the provider (as one aspect of trustworthiness) [40]. In contrast to this, it could be particularly self-references that may signal the willingness to open up and, hence, may promote a trustworthy relationship, as this could lead to reciprocal exchange of information [38]. Accordingly, there is also some evidence that a high number of self-references led to higher trust-related judgments [45].

To sum up, the use of personal reference seems to affect people’s judgments of a provider’s trustworthiness. The assumptions on the impact of personal references are ambiguous, but in the majority, the use of first-person pronouns decreased people’s perceived trustworthiness of providers, which could be caused by people’s expectation on how experts should communicate. On the basis of these thoughts, we formulated the following hypothesis:

H2: A vlogger’s use of YouTube-typical language (the use of first-person pronouns) in a Web-based video causes people to evaluate the video’s information as being less credible and the video’s vlogger as being less trustworthy compared with when vloggers use formal language (no first-person pronouns).

#### Context of Web-Based Health Videos

As previously described, the context of Web-based communication should be taken into account in addition to the content of information and the language style of information providers when investigating health information seekers’ trust-related evaluations of Web-based information and information providers. According to LET and CAT, the context of Web-based communication should impact not only how people communicate but also people’s evaluations of the interlocutors—irrespective of one’s used language style [36,37]. Hence, besides affecting users’ language use [68,69] (see also this study’s first investigation), different Web-based contexts might also individually influence people’s judgments of information and providers. The context of Web-based communication is considered norms and rules about the online media affordances, cues accompanying affordances, and how and what for these cues are used by users. As such, diverse Web-based platforms may represent different contexts of Web-based communication. However, evidence on any individual impact of Web-based platforms on trust-related evaluations is sparse [70]. Furthermore, even within a Web-based platform, some features are associated with distinguishable norms. For instance, this would be the case for an online health forum platform where a panel of experts communicates, in 1 forum thread, mainly to an audience of medical professionals and, in another forum thread, to an audience primarily of laypersons. In this context, the experts who provided the same information to medical professionals were evaluated to be more trustworthy compared with those who provided the information to laypersons [34].

In case people judge the credibility of Web-based health information and information providers differently depending on where the information came from [34,70], the communication context might serve (heuristically) as a cue for health information seekers and, hence, influence how they evaluate Web-based information. Thus, in line with approaches focusing on the *halo effect* as a phenomenon leading information seekers to biased judgments [30,88], seekers’ perceptions and evaluations of the Web-based platform might be transferred to the information found on this platform. Accordingly, a positive or negative impression of a Web-based platform would affect seekers’ judgments of information and providers in the same direction regardless of whether the platform is the source of information or rather acts as a mediator. Putting together, the contexts of Web-based communication are assumed to influence information seekers’ trust-related evaluations [30,36,37,88]. Thus, people’s impressions of Moodle and YouTube might likewise affect people’s evaluations of the Web-based health videos being presented on these platforms. As people seem to trust in YouTube rather than in Moodle (this study’s second investigation), the following hypothesis is derived.

H3: As people are more familiar and willingness to transact with YouTube compared with Moodle and this causes them to trust YouTube more than in Moodle, a Web-based video presented on YouTube causes health information seekers to evaluate the video’s information to be more credible and the video’s vlogger to be more trustworthy compared with when they evaluate the information and the vlogger of a video presented on Moodle.

#### Language Style in Specific Contexts of Web-Based Communication

In addition to studying the main effects of the context of Web-based communication and the vlogger’s language style in a Web-based video, we are also interested in whether both of these factors interact and, hence, lead people to evaluate the credibility of information and trustworthiness of vloggers differently because of a perceived appropriateness of language style, given a certain context of Web-based communication. Again, by referring to our considerations described in this study previously, the context of communication is expected to influence users’ language use within a certain context and therefore should also influence other users’ expectancies on either appropriate or not appropriate language use given this context. In turn, users’ evaluations of the appropriateness of information providers’ language use given a certain context, should also impact users’ trust-related evaluations of information and information providers [5,36,37]. Thus, even the same provider’s language style can cause differences in the quality of trust-related evaluations [76-78], as according to LET and CAT, the appropriateness of language styles given a certain context—respectively community—is also crucial for people’s evaluations.

There are some studies indicating that information seekers’ trust-related evaluations of the Web-based information and the information provider are influenced by whether people perceive the provider’s language use as either appropriate or not appropriate given a certain Web-based context. For instance, when (lay)people were exposed to nutrition information that was provided in the forum intended for medical professionals, they perceived the information which had a high amount of medical technical jargon to be more credible compared with the same information that was provided in the forum intended for laypersons. Conversely, they perceived the information that was provided in the forum intended for laypersons, which had a low amount of medical technical jargon to be more credible compared with the same information that was provided in the forum intended for medical professionals [34]. Hence, people evaluated the information to be more credible when the language was adapted to the intended audience. Similarly, in a different study, when information providers used either personalized messages (ie, messages with first-person pronouns and self-disclosure concerning provider’s health) or depersonalized messages (ie, messages without first-person pronouns and without self-disclosure), people rated the providers’ trustworthiness differently depending on whether the messages were presented on Twitter or Facebook. Although it is not clear whether 1 of the 2 language styles was expected to be more typical for either Twitter of Facebook, people rated providers who tweeted depersonalized messages on Twitter to be more competent than providers who posted with depersonalized messages on Facebook; conversely, people rated providers who posted personalized messages on Facebook to be more competent than providers who tweeted personalized messages on Twitter [62]. Nevertheless, the research on any reciprocal impact of information provider’s (appropriate) language style and the context of Web-based communication is sparse.

By considering these thoughts, we assumed that people find it more appropriate if a vlogger’s language style in a Web-based video matches people’s expected rules and norms of its platform [36,37]. More simply, a YouTube-typical language style (such as the use of first-person pronouns: this study’s first investigation) might be perceived as appropriate on YouTube. Conversely, as Moodle is a learning platform that includes materials about academic issues, low self-disclosure (such as the nonuse of first-person pronouns in formal language) might be seen as more professional and appropriate on Moodle [41]. Hence, we derived the following hypothesis on an interaction effect:

H4: A Web-based video presented on YouTube causes people to evaluate the information to be more credible, the vlogger to be more trustworthy, and the vlogger’s language use to be more accommodative when the vlogger uses YouTube-typical language than formal language. Instead, a Web-based video presented on Moodle causes people to evaluate the information to be more credible, the vlogger to be more trustworthy, and the vlogger’s language use to be more accommodative when the vlogger uses formal rather than YouTube-typical language. As it is assumed that people evaluate the credibility of information and trustworthiness of the information provider depending on their perception of how appropriately a vlogger’s language accommodates the community of a certain Web-based platform, participants were also asked to rate how they perceived the accommodation of the vlogger’s language use.

## Methods

### Investigation 1

#### Procedures

To identify characteristics of users’ language styles that are typical for either Vimeo or YouTube, we systematically selected health videos from each platform based on the most relevant hits of keyword searches. On YouTube, searches were conducted on July 26, 2016 (for *health*), and July 28, 2016 (for *diet*); on Vimeo, searches were conducted for both *health* and *diet* on August 1. For each search term, the first most relevant 50 results were collected (the option of filtering by the *most relevant* was selected). Videos were excluded if they were longer than 10 min as the duration of about half of the 332,382 investigated Web-based videos in connection to YouTube’s traffic were between 3 and 5 min [107]. In addition, videos were excluded for the following reasons: if the video was an advertisement (eg, movie trailers, brands, and products; except for explanations by health institutions), if the video did not have spoken content, if more than 1 person was speaking, or if the spoken language was not English. None of the videos appeared under both search terms. After exclusion and deletion, 36 videos remained (25 videos from YouTube and 11 from Vimeo). Furthermore, the following characteristics were recorded: original rank of video, the presence of a protagonist, duration, declared or apparent topic, or aim of the video (Multimedia Appendix 1).

#### Analyses

The transcripts of videos were analyzed with Linguistic Inquiry and Word Count (LIWC2007) by Pennebaker Conglomerates, Inc [108], to identify differences in the number and quality of pronouns. LIWC2007 is a computer program that analyzes the absolute and relative word frequency of texts based on included dictionaries and predefined categories of words. Transcribed texts of videos were implemented to LIWC2007, and analyses were conducted to identify differences in the relative number of pronouns used in the transcripts of the YouTube and Vimeo videos. Therefore, we used the LIWC’s dictionary, including all singular and plural forms of first-person, second-person, and third-person pronouns (included in dictionary but irrelevant for this investigation). We analyzed the number of first-person and second-person pronouns relative to the overall word frequency of a transcript and tested differences between the extent of pronoun use on both platforms by conducting a Welch analysis of variance that is relatively robust against unequally distributed variances in dependent variables among Web-based platforms.

### Investigation 2

#### Procedures

We conducted a Web-based survey by using *Questback’s EFS Survey* [109]. Through an automatic balanced randomization, participants were randomly assigned the order in which they answered questions about the 2 platforms, either first about YouTube and then about Moodle, or vice versa. A total of 75 participants first answered items concerning Moodle, whereas 76 participants first answered items concerning YouTube. In the survey, both platforms were briefly introduced to guarantee that participants were equally familiar with each of the platform’s purposes. YouTube was introduced as follows: “The YouTube video platform is mainly used for viewing, providing, commenting and sharing videos, and it is the largest and most comprehensive Web-based video portal.” Moodle was introduced as follows: “The learning platforms of Moodle serve educational institutions (eg, universities) mainly to provide learning opportunities. In the Web-based courses on Moodle (which often accompany in-person courses), lecturers and learners can upload learning materials such as texts and learning videos.”

#### Participants

People were invited to take part in the survey through a link on Web-based platforms run by several German universities. A total of 151 participants aged between 18 and 61 years (mean 30.21 [SD 9.78]) took the survey. Of 151, 88 participants stated that they are studying, 54 participants declared to be employed, and 148 participants declared German to be their first language. The participants’ self-reported weekly computer usage was, on average, 28.91 hours (SD 16.44), and their average weekly internet usage was 24.35 hours (SD 13.31). Furthermore, participants rated the frequency (from 1: daily to 5: never) of watching videos on YouTube, in online media libraries, and on streaming services to be, on average, 2.34 (SD 0.93)—meaning *several times a month*.

#### Measures

Participants answered 14 items on a 7-point Likert scale from 1 (I strongly disagree) to 5 (I strongly agree) once for Moodle and once for YouTube. Items were adapted from the scale *Trust in Online Firms* [86]. On the basis of a study by Mayer, Davis, and Schoorman [25], 2 items for ability, integrity, and benevolence and 1 item for the platform’s overall trustworthiness were used to assess the trustworthiness of the platform (eg, *Moodle or YouTube has the skills and expertise to provide videos in an expected manner* or *Moodle (or YouTube)* *makes good-faith efforts to address most customer concerns*). Moreover, people’s *willingness to transact* was assessed using 3 items (eg, *I watch videos on Moodle or YouTube* or *I am likely to utilize the services provided by Moodle or YouTube* *)* [86], and people’s perceived familiarity with the platform was assessed by using 4 items (eg, *I am familiar with searching for videos on Moodle or YouTube*) [86].

### Investigation 3

#### Exclusion Criteria and Participants

An analysis of power (assuming 1−beta=.80; Cohen f^2^=.0625) was used in advance to determine that a sample size of N=113 would be sufficient assuming a small effect size. Using a mailing list of a large German university, participants were invited to participate in a Web-based survey and received a €8 voucher as reimbursement. In total, 128 participants completed the survey, in which people who were interested in participating were automatically excluded by the survey programming in the following cases: if they stated that they are studying or had studied medicine, food chemistry, or nutrition science (as it was assumed that their high prior knowledge would influence credibility and trustworthiness judgments) and if they stated that they are studying psychology or chemistry and pharmacy (it was possible that they might know the psychology lecturer acting as the nutrition vlogger in the experiment video or they might suspect the chemistry course in the Moodle condition to be artificial created). Furthermore, survey access was automatically denied when using a mobile phone (thus, the screen size of used devices was controlled). All participants specified at the end of the survey that they did want to provide their data for research purposes. Furthermore, 2 participants were excluded from data analysis because they stated that they knew the psychology lecturer acting as the vlogger. In addition, 2 other participants were excluded because their time to complete the survey took more than 1 standard deviation above the overall mean duration of all participants (mean 40.5 min [SD 130.55]).

Although 20 participants did not recognize the video platform, it is unclear whether these participants might have unconsciously perceived platforms. Nonetheless, we decided to include these participants’ data in analysis. Therefore, 124 participants (72 female) aged 18 to 48 years (mean 22.65 [SD 3.45]) were included in data analysis, with 30 to 32 participants in each experimental condition. The mean duration of time to complete the survey was 26.58 min (SD 13.68). In addition, 119 participants stated that they were students, and 121 participants declared German to be their first language (the other 3 participants had been speaking German for at least 17 years). Every week, participants used the computer, on average, for 27.89 hours (SD 17.53) and the internet for 34.76 hours (SD 20.83). Furthermore, participants rated their frequency (from 1: daily to 5: never) of watching videos on YouTube, in online media libraries, on streaming services, and learning platforms to be, on average, 2.96 (SD 0.60)—meaning *several times a month*, and watching videos specifically on learning platforms was, on average, 3.91 (SD 1.13)—meaning *less than once a month*. Moreover, all participants reported that their prior knowledge and motivation to learn about the topic *health and nutrition* (from 1: I disagree to 5: I agree) was, on average, 3.41 (SD 0.83); therefore, they were rather interested and cognizant of the topic.

#### Design

Participants were randomly assigned to 1 of the 4 cells of the 2 × 2 between-subject factors, which were context of Web-based communication (YouTube vs Moodle) and language style of the vlogger (YouTube-typical vs formal language), to examine the impact of both factors on information credibility, the vlogger’s trustworthiness, and the perceived language accommodation of vloggers. Furthermore, participants were confronted with all manifestations of the 1 × 3 within-subject factor, which was uncertainty of information, meaning that they saw videos containing 2 of the following explanations: evidence for the nutrition myth (confirming), evidence against the nutrition myth (disconfirming), or unclear evidence that indicated the scientific findings neither confirm nor disconfirm the nutrition myth.

For varying the Web-based communication contexts, we manipulated the video’s platform using 2 aspects that introduced where the video came from. First, to ensure a plausible comparison between Moodle and YouTube, participants read an email by a nutritional science lecturer who linked the video with a reference either to YouTube or Moodle (Multimedia Appendix 2). Accordingly, this introductory material explained that the videos are intended to supplement a nutrition course. This introduction was given to prevent participants from being suspicious, which might have been the case if they were told to watch a video on Moodle without any prior explanation. After reading the email by the nutrition science lecturer, participants saw the video embedded in previously created screenshots according to the 2 conditions of either Moodle (an academic learning platform that allows for the exchange of material to supplement an academic course called *learnweb* for the German university where participants were recruited) or YouTube (Multimedia Appendix 3). The language used in videos was realized by a vlogger who used either YouTube-typical or formal language.

#### Procedure

Participants completed a Web-based survey via the tool Questback’s EFS Survey [109]. In the beginning, a test page ensured that participants’ devices could properly display the videos. After answering questions regarding demographic and control variables, participants were randomly assigned to the experimental conditions via a balanced randomization. According to the experimental conditions, they first read a lecturer’s email with references to a video on either Moodle or YouTube (Multimedia Appendix 2). To ensure the participants paid attention to this introduction, they were able to continue after at least 25 seconds. Hereupon, participants viewed the video about nutrition myths spoken either in YouTube-typical or formal language. Both videos included 2 explanations that either confirmed or disconfirmed or (through unclear evidence) neither confirmed nor disconfirmed the nutrition myths. Once again, according to the experimental condition, videos were embedded in screenshots showing either a Moodle or YouTube surface. Continuing was allowed after at least 5 min to ensure that participants did not skip the video. After watching the video for the first time, participants rated the vlogger’s trustworthiness, the vlogger’s accommodation, and the control variables regarding the video’s relevance in relation to the topic of nutrition. Every video sequence for each of the nutrition myths was shown a second time, which enabled participants to assess the credibility of information and control variables regarding feeling of knowing (3 items) for each of the nutrition myths individually. At the end, by answering open questions, participants were asked to assess the language of the vlogger, to remember the platform from which the video stemmed (manipulation check), and to state their generally used criteria when deciding whether to watch videos on a specific platform (explorative).

#### Experimental Materials

Videos included explanations of scientific findings with respect to 6 typical nutrition myths. All nutrition myths (in each video presented in this order: *coffee and dementia, cola and pretzel sticks, too many diet beverages leading to diabetes, the healthiness of low salt diet, harmfulness of too many eggs, healthy nutrition and cancer*) resulted from Web-based searches for frequent and typical nutrition myths in online forums. Although all explanations reflect the current scientific findings about related topics [110-116], explanations summarized the scientific evidence as if it speaks for, against, or neither for nor against the nutrition myths.

In the following, the explanations for the myths about coffee and dementia and light beverages and diabetes are summarized, whereby both conclusions were not clear as the scientific evidence was summarized such that it speaks neither for nor against the myths. The first vlogger’s explanation related to the nutrition myth about *coffee and dementia* that claims coffee inhibits the risk of developing dementia. The vlogger explained that coffee, in fact, has all sorts of positive and negative physical effects, and that coffee contains the substance caffeine, which like other building blocks of our DNA has a stimulating effect on the brain. The vlogger further reported that there are long-term studies showing coffee consumption reduces the risk of developing Alzheimer disease by 16%, but that it is still unclear whether only caffeine or other ingredients and factors are responsible for that. Another vlogger’s explanation related to the myth of *too many diet beverages leading to diabetes*. The vlogger said that it is well known that in the long run, people are more likely to get diabetes if they often drink beverages with artificial sweeteners—compared with people who drink such drinks rarely or never. The vlogger said further that it is uncertain whether the sweeteners are responsible for the increase of diabetes, and that people instead might prefer to drink light beverages when they have a tendency to be overweight.

The explanations for the myths about cola and pretzel sticks and harmfulness of too many eggs contained conclusions that disconfirm the myths, as the scientific evidence was summarized such that it speaks against the myths. The vlogger explained underlying scientific results related to the myth of *cola and pretzel sticks*, which are said to help stop diarrhea. She explained that cola consists mostly of sugar, which leads to liquid being removed from the body. Further she explained that cola contains a lot of caffeine, which has different effects: it makes you awake, but stimulates your kidneys, which leads to more potassium loss. She concluded that even the popular mixture *cola with pretzel sticks* does not change anything because if one eats salty pretzel sticks, they only contain saline and, therefore, are not able to remedy the potassium deficiency. Similarly, the vlogger concluded that the myth regarding the *harmfulness of too many eggs* as not scientifically confirmed. According to this myth, the vlogger said that the yolk of an egg is not only quite rich in fat but also contains a lot of cholesterol. She explained that a medium-sized egg of 60 g supplies 270 mg of the fat, but there is no correlation between the risk of cardiovascular disease and the consumption of eggs in any large-scale study. Hence, she explained, if people get a lot of cholesterol in the form of eating eggs, this does not necessarily mean that it is harmful—especially because cholesterol also has positive properties. She mentioned that this is different for diabetics because there are some findings that indicate more cardiovascular disease for diabetics who ate lot of eggs.

Finally, the vlogger’s explanations related to the myths of healthy nutrition and cancer and the healthiness of low salt diet concluded that the myths can be confirmed from a scientific perspective. In terms of *healthy nutrition and cancer*, the vlogger explained that there are many indications that for people who eat in a balanced way, move enough, and have a normal body weight have a lower risk of developing cancer. She further explained that cardiovascular diseases, obesity, hypertension, and sugar disease are less frequent for people who eat healthy, and there are studies showing that diet plays an essential role in preventing colorectal and breast cancer. She mentioned that for other types of cancer, for instance, esophageal cancer, there are currently only a few indications that may indicate a connection. Moreover, she said that what is considered as healthy diet varies according to the state of knowledge, and there are no broad-brush recipes, and that the latest recommendations focus not only on what should be eaten but also on how much. Hence, she concluded that the energy balance—of what you eat and how much of it—is just as crucial as eating whole grain products, legumes, vegetables, and fruit. She said that people should only consume energy-rich foods, sugary drinks, red meat, and salty foods in moderation. In terms of *the healthiness of a low salt diet*, the vlogger explained that there is a lot of evidence showing that a low-salt diet affects blood pressure, which is important because high blood pressure will lead to heart disease and enlarged vessels in the long term. She further explained that, therefore, the World Health Organization recommends people to reduce their daily intake of common salt to about 6 g. This is supposed to reduce systolic blood pressure (the upper number in a blood pressure reading) by 5 to 6 mm and reduce the diastolic blood pressure (the lower number in a blood pressure reading) by 1 to 3 millimeters; this should have the same effect as losing weight.

To construct the YouTube-typical version, we adopted criteria developed by Mayer et al [106] and added some typical characteristics of language style which we extracted from 37 transcribed YouTube and Vimeo videos—more first-person pronouns and self-references in YouTube videos (this study’s first study). Thus, we formulated a second version of the vlogger’s language style for each video that replaced the personal pronouns (eg, *you*) with more formal words such as *the* and also omitted typical YouTube characteristics (eg, *I*). In the YouTube-typical language condition, texts contained more words because of personalization, so the video lengths differ by 63 seconds. Both videos were created and hence are identical regarding content, the vlogger, and design (videos were also used in another experiment by the authors [35]). In this vein, the information providers in both videos were comparable, as a psychology lecturer was acting the information provider in the same way. Furthermore, we did not inform participants about vlogger’s expertise explicitly. This might be important to consider, as people also judge the trustworthiness and the credibility of information based on the information providers’ expertise or other relevant characteristics [117].

To manipulate the platform of video, screenshots were produced showing either a Moodle or a YouTube surface. Therefore, frames of real YouTube videos were used, underlying titles were created, and the video about nutrition science was embedded. The Moodle screenshot was designed by creating an academic course about nutrition science where the embedded video about nutrition myths was uploaded (Multimedia Appendix 3).

#### Dependent Measures

##### Credibility of Information

Participants indicated on 1 item whether they agree with the given information, and they judged the information credibility on 5 additional items adopted by a measurement of trust in journalism [118]. Overall, these 6 items (5-point Likert scale, with 1: I strongly disagree to 5: I strongly agree) yielded internal consistencies for each nutrition myth between Cronbach alpha=.83 and Cronbach alpha=.86.

##### Trustworthiness of the Vlogger

Vlogger’s epistemic trustworthiness was assessed with the Muenster Epistemic Trustworthiness Inventory (METI) [16]. METI is composed of 3 subscales: *Expertise* reflects people’s perception of an expert as truly knowledgeable, intelligent, and highly trained in her domain (6 items). *Integrity* reflects people’s perception of an expert’s good character, her values, and her as a person, which is acting in line with principles (4 items). *Benevolence* reflects people’s perception of an expert’s orientation toward others or society and represents whether an expert acts in accordance with the interest of others (4 items). Participants rated these items on 7-point semantic differentials (eg, 1: competent to 7: incompetent). Internal consistencies yielded Cronbach alpha=.91 for the 6 competence-related items, Cronbach alpha=.86 for the 4 integrity-related items, Cronbach alpha=.85 for the 4 benevolence-related items, and Cronbach alpha=.93 for the aggregated score of all 14 items.

##### Perceived Accommodation of the Vlogger’s Language Style

Participants assessed how they perceived the vlogger’s accommodation with an adaption of the Recipient Orientation Scale (ROS) [119]. *Audience design* reflects how people perceive the willingness of a vlogger to adapt to the audience (eg, *the person can imagine how it is to know little about this topic*). *Evaluation* reflects how people perceive the motivation of an expert to explain herself (eg, *the person cares about mediating her expertise*). *Subjective comprehension* reflects people’s self-reported understanding about the topic (eg, *I understood the content*). Overall, internal consistencies yielded Cronbach alpha=.83 for the 9 items related to audience design, Cronbach alpha=.82 for the 4 items related to evaluation, Cronbach alpha=.77 for 2 items related to subjective comprehension, and Cronbach alpha=.87 for the aggregated score of all 15 items. For all items, a 5-point Likert scale from 1 (I strongly disagree) to 5 (I strongly agree) was used.

##### Control Variables

Participants were asked to report their prior knowledge and motivation to learn about the topic (4 items; eg, *I am familiar with the topic Health and Nutrition*) before they were randomly assigned to the experimental conditions. After watching the video 1 time, we assessed how participants perceived the relevance of videos in terms of the topic of nutrition (3 items; eg, *the video is relevant to a video about Health and Nutrition*). In addition, we assessed subjective familiarity, subjective complexity, and interest (3 items *feeling of knowing*) for each of the myths. For all items, a 5-point Likert scale from 1 (I strongly disagree) to 5 (I strongly agree) was used. Finally, a manipulation check asked *How do you evaluate the language of the person?* and *On what platform was the video?*

#### Analyses

We preliminarily analyzed differences between groups of our 4 conditions regarding any expected control variables. A multivariate variance analysis revealed no differences regarding the demographic variables of age, gender, and frequency of using the internet and a computer and the frequency of watching Web-based videos, participants’ prior knowledge, and their *feeling of knowing* in terms of the nutrition myths (all *F*_3,119_≤1.62; *P* ≥.19; *η_p_^2^* ≤0.04). However, considering the significant Pearson correlations between age and trustworthiness, age and vlogger’s accommodation (all *r* ≤|.231|; *P* ≥.01) between perceived relevance of the videos and all dependent variables (all from *r*=|.197| to *r*=|.533|; *P* ≤.03) and between feeling of knowing and all dependent variables (all from *r*=|.184| to *r*=|.420|; *P* ≤.02) these control variables should be included in the main analysis, as those variables might explain variance of the dependent variables. Pearson correlations between all other control variables and dependent variables were not significant (all from *r*=|.001| to *r*=|.165|; *P* ≥.07). Taking these results together with the results of the multivariate variance analysis, which revealed no differences between experimental conditions regarding any control variables, none of these uncorrelated variables were included as control variables in the main analysis. Correlations for dependent and control variables for the third investigation are provided in Multimedia Appendix 4.

In addition, we analyzed whether the frequencies of participants who stated that they did not remember the video platform differed between conditions. Therefore, a chi-square test was used to compare the video platform and the manipulation check (recognized platform). Cell frequencies were between 2 and 58. Results showed a significant difference in the frequency between the video platform and the manipulation check (χ²_1_=14.1; *P*<.001; φ=.337). Of the 20 participants who stated that they did not remember the platform, 18 participants were in the condition that saw the video on the Moodle platform.

Moreover, we asked for the criteria participants used when deciding whether to watch videos on a specific platform, and we categorized their replies into aspects regarding (1) technical features of the platform, (2) quality features of the platform, (3) familiarity with the platform, (4) trust in the platform for a specific purpose, (5) others’ recommendations of the platform, (6) the protagonist in the video, (7) the scientific nature of content, (8) the interest in content, (9) technical features of the video, (10) none, and (11) not specified. A chi-square test was used to compare participants’ assignment to the video platform condition and the frequencies of these categories. Cell frequencies were between 2 and 43. Results showed no significant differences in frequencies between the video platform they were assigned to and these categories (χ²_10_=6.7; *P=*.75; φ=.010). Hence, the number of participants who stated that they use criteria belonging to the above-mentioned categories was not different between the video platform conditions of Moodle and YouTube.

Setting the global alpha level of .05, we performed a multivariate variance analysis with the video platform and the language style as between-subject factors and the trustworthiness and the vloggers’ accommodation as the dependent measures. Furthermore, another variance analysis with repeated measures was conducted in terms of the dependent measure credibility, again including the video platform and language style as the between-subject factors and the uncertainty of information as the within-subject factor. Myths were composited in terms of their overall conclusion and, therefore, in terms of whether the explanations and underlying scientific findings were (1) not clear (2) conflicting with, or (3) consistent with the myth. Hence, for each participant, 3 averaged values of credibility ratings were analyzed as within-subject measures because participants were asked to rate each of the myths on the same items each time they watched the video. As we conducted 2 analysis for the testing of hypotheses in 1 sample, we adjusted local alpha levels and set the local alpha level^1^ of .025 and the local alpha level^2^ of .05, according to the Bonferroni-Holm adjustment. All tests were 1-sided.

## Results

### Investigation 1

Results showed that the duration of videos from YouTube (mean 339.6 seconds [SD 134.32]) was longer than from Vimeo (mean 166.18 seconds [SD 116.33]; *F*_1,22_=15.4; *P*=.001). In addition, the total number of spoken words in videos from YouTube (mean 981.24 [SD 547.61]) was higher than the total number of words in videos from Vimeo (mean 320.18 [SD 175.82]; *F*_1,32_=29.56; *P*<.001). It is plausible that the higher word count from YouTube videos might be related to longer durations of the YouTube videos.

On average, videos from YouTube contained 50.3 (SD 59.25) first-person singular pronouns (me, I, and my) of 981.24 words. On average, videos from Vimeo contained 6.7 (SD 10.01) first-person singular pronouns of 320.18 words. The analysis of variance revealed that the relative number of first-person singular pronouns on YouTube (mean 4.4% [SD 3.94]) was higher than on Vimeo (mean 1.47% [SD 2.12]; *F*_1,32.35_=8.17; *P*=.007). On average, videos from YouTube contained 34.84 (SD 26.78) second-person singular pronouns (you, your, and thou) of 981.24 words; on average, videos from Vimeo contained 11.73 (SD 6.77) second-person singular pronouns of 320.18 words. The analysis of variance revealed no differences regarding the use of second-person pronouns in YouTube (mean 3.58% [SD 2.03]) and Vimeo (mean 4.41% [SD 2.95]; *F*_1,14.36_=.714; *P*=.41).

### Investigation 2

Paired-sample *t* tests revealed differences for people’s trust in Web-based platforms (score of all 14 items) and for people’s willingness to transact and people’s familiarity with the Web-based platform; results showed lower trust in Moodle (mean 4.28 [SD 1.41]) than in YouTube (mean 5.25 [SD .84]), less willingness to transact with Moodle (mean 3.81 [SD 1.85]) than with YouTube (mean 5.91 [SD 1.02]), and less familiarity with Moodle (mean 3.82 [SD 1.81]) than with YouTube (mean 5.84 [SD .87]; all *t*_150_≤−9.63; all *P* ≤.001). There was no difference for the items related to trustworthiness (ability, benevolence, integrity, and overall trustworthiness) of Moodle (mean 4.74 [SD 1.23]) compared with YouTube (mean 4.63 [SD 1.18]; *t*_150_=1.06; *P*=.29), meaning that, overall, participants reported that they trust in YouTube more than in Moodle, although their answers regarding the items called *trustworthiness* did not differ between Moodle and YouTube. In particular, participants’ familiarity and willingness to transact with YouTube were higher compared with Moodle. A post-hoc analysis was conducted to identify whether participants’ self-reported frequency of watching Web-based videos explains additional variance of the dependent variables. This analysis, indeed, reveals that participants’ self-reported frequency of watching Web-based videos on YouTube (1 item) had significantly impacted participants’ trust in Moodle and YouTube (both *F*_1,149_≥4.75; *P* ≤.03; η_*p*_^2^≥0.03). Furthermore, participants’ frequency of watching Web-based videos in online media libraries (1 item) had significantly impacted participants’ trust in Moodle (*F*_1,149_=10.8; *P*=.001; η_*p*_^2^=0.07), but not participants’ trust in YouTube (*F*_1,149_=.395; *P*=.53). Finally, participants’ frequency of watching Web-based videos on streaming services (1 item) had significantly impacted participants’ trust in YouTube (*F*_1,149_=13.86; *P*=.001; η_*p*_^2^=0.09), but not participants’ trust in Moodle (*F*_1,149_=.353; *P*=.06; η_*p*_^2^=0.023).

### Investigation 3

#### Credibility of Information

**Figure 1:**
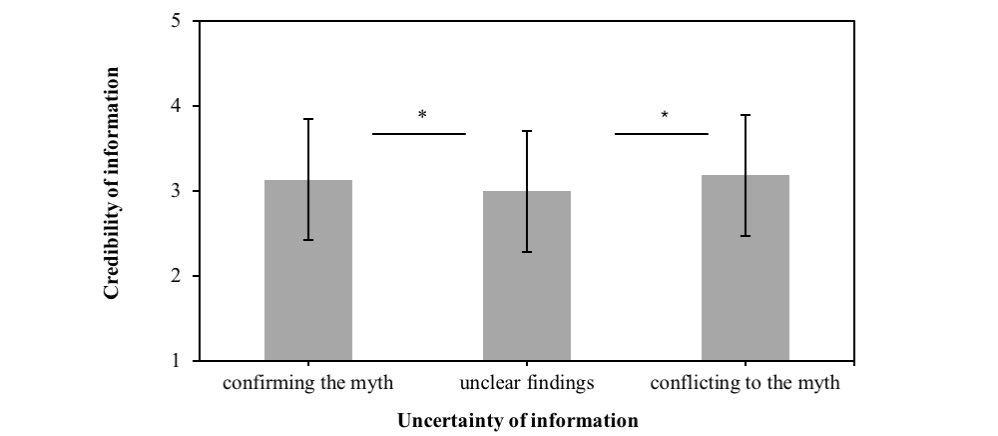
Participants’ credibility judgments in terms of the uncertainty of information in the experiment (within subjects). The following covariates were included in the analysis due to the following values: age=22.65, relevance of video=3.36, feeling of knowing=3.55.

The variance analysis with repeated measures yielded no between-subject main effect of video platform (*F*_1,117_=2.40; *P=*.06; η_*p*_^2^=0.02), and language style (*F*_1,117_=.12; *P=*.37; η_*p*_^2^=0.001), for participants’ credibility judgments. Furthermore, there was no significant interaction of language style and video platform (*F*_1,117_=2.06; *P=*.08; η_*p*_^2^=0.02). Furthermore, the analysis yielded a significant effect for uncertainty of information (*F*_2,116_=9.109; *P*<.001; η_*p*_^2^=0.136). Post hoc group comparisons showed that explanations with unclear findings (*coffee and dementia* and *too many diet beverages leading to diabetes*) were judged to be significantly less credible than explanations conflicting with the myth (*cola and pretzel sticks* and *harmfulness of too many eggs*; *d*=|1.9|, SE 0.05; 95% CI 0.08-0.30; *P*<.001). Moreover, these explanations with unclear findings were judged to be significantly less credible than explanations confirming the myth (*healthy nutrition and cancer* and *the healthiness of low salt diet*; *d*=|1.4|, SE 0.05; 95% CI 0.25-0.03; *P=*.004). Furthermore, comparisons showed that those explanations conflicting with the myth (*cola and pretzel sticks* and *harmfulness of too many eggs*) and those confirming the myth (*healthy nutrition and cancer* and *the healthiness of low-salt diet*) did not lead to significantly different credibility ratings (*d*=|0.05|, SE 0.04, 95% CI 0.06-0.16; *P=*.77). Figure 1 shows credibility judgments for the uncertainty of information.

#### Trustworthiness of the Vlogger

The multivariate analysis revealed no main effects of video platform for all trustworthiness measures (all *F*_1,117_≤.18; *P* ≥.34; η_*p*_^2^≤0.002). Furthermore, there were no main effects of language style for the METI score and the subscales competence and integrity (all *F*_1,117_≤0.86; *P* ≥.18; η_*p*_^2^≤0.007). However, there was a main effect of language style for the subscale benevolence (*F*_1,117_=3.41; *P=*.03; η_*p*_^2^=0.028). That is, YouTube-typical language led to higher benevolence ratings (mean 2.63 [SD 0.93]) than formal language (mean 2.94 [SD 0.91]). Moreover, the multivariate analysis yielded no significant interactions of language style and video platform for all trustworthiness measures (all *F*_1,117_≤1.18; *P* ≥.14; η_*p*_^2^≤0.01).

#### Perceived Language Accommodation by Vlogger

**Figure 2:**
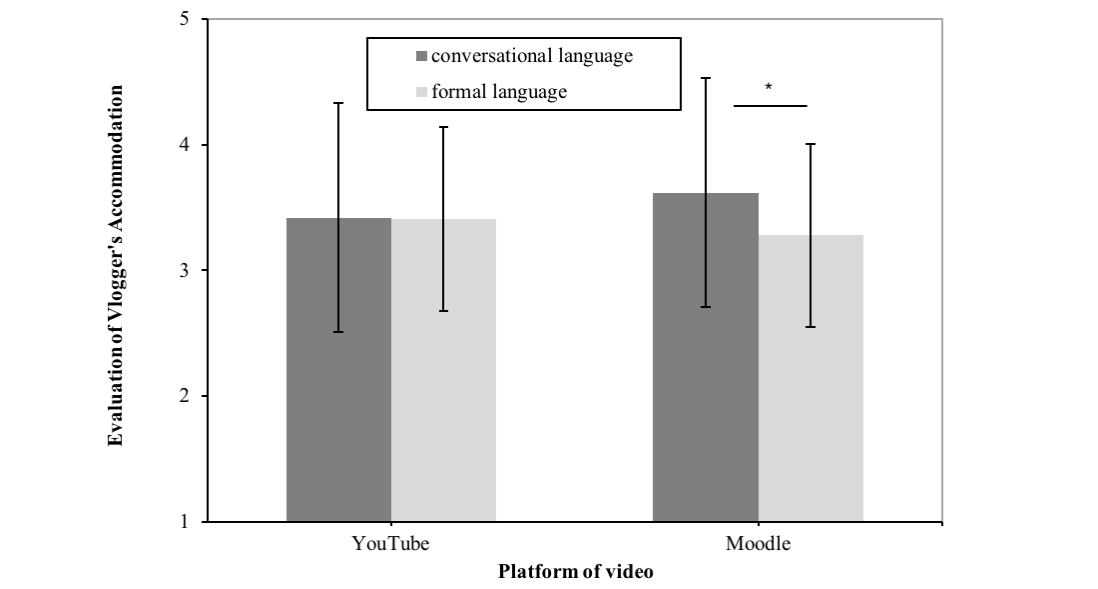
Participants’ evaluations on the Evaluation subscale (ROS Evaluation) for the factors video platform and language style in the experiment. The following covariates were included in the analysis due to the values: age=22.65, relevance of video=3.36, feeling of knowing=3.55.

The multivariate analysis revealed no main effects of video platform for the perceived accommodation of vlogger’s language (all *F*_1,117_≤1.29; *P* ≥.13; η_*p*_^2^≤0.011). However, analysis yielded a main effect of language style for the overall score of the recipients orientation scale (*F*_1,117_=8.41; *P=*.002; η_*p*_^2^=0.07), and its subscale audience design (*F*_1,117_=10.59; *P*<.001; η_*p*_^2^=0.08), with more ascribed vlogger’s accommodation when she used YouTube-typical language compared with formal language. The analysis revealed no significant main effect of language style on the subscales evaluation and subjective comprehension (both *F*_1,117_≤1.31; *P* ≥.13; η_p_^2^≤0.011). Furthermore, analysis yielded a disordinal significant interaction effect of platform of video and language style for the subscale evaluation (*F*_1,117_=5.40; *P=*.01; η_*p*_^2^=0.04). That is, more ascribed accommodation in Moodle for the vlogger who used YouTube-typical language compared with formal language (*d*=|.43|, SE 0.17; *P=*.04, Bonferroni post-hoc analysis). Conversely, in YouTube, the vlogger’s accommodation for either formal or YouTube-typical language was not judged differently (*d*=|.15|, SE 0.18; *P=*.50). Similarly, none of the other pairwise comparisons of experimental conditions yielded significant differences of means (all d≤|.34|, SE 0.18; *P* ≥.17). Figure 2 illustrates this interaction for the factors video platform and language style on the subscale evaluation.

Table 1 shows descriptive values for the factors’ *platform of video* and *language style* for the dependent variables, credibility of information, trustworthiness, and vlogger’s language accommodation. In addition, Table 2 shows values for the multivariate analysis of variance including the control variables age, relevance of video, and feeling of knowing and the factors’ platform of video and language style for the dependent variables’ trustworthiness and vlogger’s language accommodation.

**Table 1:** Descriptive values for the factors’ platform of video and language style of vlogger for the dependent variables trustworthiness (eg, 1: competent to 7: incompetent), credibility of information, and vlogger’s language accommodation (1: I strongly disagree to 5: I strongly agree).

Dependent variables and platform of video			YouTube-typical language, mean (SD)	Formal language, mean (SD)	Total, mean (SD)
**Trustworthiness of vlogger**					
	**METI^a^ score**				
		Moodle	2.80 (1.01)	2.77 (0.77)	2.79 (0.89)
		YouTube	2.78 (0.83)	2.97 (0.79)	2.88 (0.81)
		Total	2.79 (0.92)	2.87 (0.78)	2.83 (0.85)
	**METI competence**				
		Moodle	3.05 (1.21)	2.94 (1.07)	2.99 (1.13)
		YouTube	3.12 (1.00)	3.01 (.98)	3.06 (0.98)
		Total	3.08 (1.11)	2.97 (1.02)	3.03 (1.06)
	**METI integrity**				
		Moodle	2.59 (1.07)	2.49 (0.73)	2.54 (0.91)
		YouTube	2.42 (0.91)	2.83 (0.73)	2.63 (0.84)
		Total	2.51 (0.99)	2.65 (0.75)	2.58 (0.88)
	**METI benevolence**				
		Moodle	2.64 (1.02)	2.80 (0.80)	2.72 (0.91)
		YouTube	2.61 (0.85)	3.08 (1.02)	2.84 (0.96)
		Total	2.63 (0.93)	2.94 (0.91)	2.78 (0.93)
**Vlogger’s language accommodation**					
	**ROS^b^ score**				
		Moodle	3.84 (0.58)	3.53 (0.54)	3.69 (0.58)
		YouTube	3.81 (0.50)	3.61 (0.58)	3.71 (0.55)
		Total	3.83 (0.54)	3.57 (0.56)	3.70 (0.56)
	**ROS audience design**				
		Moodle	3.91 (0.61)	3.53 (0.62)	3.72 (0.64)
		YouTube	3.94 (0.45)	3.67 (0.67)	3.81 (0.58)
		Total	3.92 (0.54)	3.60 (0.64)	3.76 (0.61)
	**ROS evaluation**				
		Moodle	3.62 (0.94)	3.28 (0.79)	3.45 (0.88)
		YouTube	3.42 (0.88)	3.41 (0.67)	3.41 (0.78)
		Total	3.52 (0.91)	3.34 (0.73)	3.43 (0.83)
	**ROS subjective comprehension**				
		Moodle	3.98 (0.79)	4.02 (0.69)	4.99 (0.73)
		YouTube	4.02 (0.79)	3.73 (0.76)	3.88 (0.78)
		Total	4.00 (0.78)	3.88 (0.73)	3.94 (0.76)
**Credibility of information**					
	**Unclear**				
		Moodle	3.21 (0.83)	3.12 (0.61)	3.16 (0.73)
		YouTube	3.25 (0.69)	3.17 (0.67)	3.21 (0.68)
		Total	3.23 (0.76)	3.14 (0.64)	3.18 (0.70)
	**Conflicting**				
		Moodle	3.08 (0.84)	2.94 (0.64)	3.01 (0.74)
		YouTube	2.89 (0.67)	3.07 (0.67)	2.98 (0.67)
		Total	2.99 (0.76)	3.00 (0.65)	3.00 (0.71)
	**Confirming**				
		Moodle	3.07 (0.79)	3.05 (0.76)	3.06 (0.77)
		YouTube	3.16 (0.64)	3.27 (0.66)	3.21 (0.65)
		Total	3.11 (0.72)	3.15 (0.72)	3.13 (0.71)

^a^METI: Muenster Epistemic Trustworthiness Inventory.

^b^ROS: Recipient Orientation Scale.

**Table 2:** Multivariate analysis of variance including the factors’ platform of video and language style of vlogger and the control variables age, relevance of video, and feeling of knowing for the dependent variables’ trustworthiness and vlogger’s language accommodation.

Source of variance		*df*	*F*	*P* value	η_*p*_^2^
**Age**					
	METI^a^ score	1	4.631	.02	0.038
	METI competence	1	7.269	.004	0.058
	METI integrity	1	2.281	.07	0.019
	METI benevolence	1	0.616	.22	0.005
	ROS^b^ score	1	8.807	.002	0.07
	ROS audience design	1	5.722	.009	0.047
	ROS evaluation	1	10.312	.001	0.081
	ROS subjective comprehension	1	0.011	.46	0
**Relevance of video**					
	METI score	1	22.332	<.001	0.16
	METI competence	1	25.078	<.001	0.177
	METI integrity	1	15.618	<.001	0.118
	METI benevolence	1	6.627	.001	0.054
	ROS score	1	15.748	<.001	0.119
	ROS audience design	1	6.216	.007	0.05
	ROS evaluation	1	23.785	<.001	0.169
	ROS subjective comprehension	1	1.72	.1	0.014
**Feeling of knowing**					
	METI score	1	6.694	.001	0.054
	METI competence	1	5.175	.01	0.042
	METI integrity	1	5.962	.008	0.048
	METI benevolence	1	3.559	.03	0.03
	ROS score	1	21.529	<.001	0.155
	ROS audience design	1	16.767	<.001	0.125
	ROS evaluation	1	11.107	.001	0.087
	ROS subjective comprehension	1	4.627	.02	0.038
**Platform of video**					
	METI score	1	0	.5	0
	METI competence	1	0.1	.38	0.001
	METI integrity	1	0.003	.48	0
	METI benevolence	1	0.182	.34	0.002
	ROS score	1	0.583	.23	0.005
	ROS audience design	1	1.289	.13	0.011
	ROS evaluation	1	0.158	.35	0.001
	ROS subjective comprehension	1	0.699	.2	0.006
**Language style**					
	METI score	1	0.157	.35	0.001
	METI competence	1	0.861	.17	0.007
	METI integrity	1	0.721	.2	0.006
	METI benevolence	1	3.412	.03	0.028
	ROS score	1	8.409	.002	0.067
	ROS audience design	1	10.587	<.001	0.083
	ROS evaluation	1	1.308	.13	0.011
	ROS subjective comprehension	1	0.749	.19	0.006
**Platform of video X language style**					
	METI score	1	0	.49	0
	METI competence	1	0.83	.18	0.007
	METI integrity	1	1.177	.14	0.01
	METI benevolence	1	0.252	.31	0.002
	ROS score	1	2.931	.05	0.024
	ROS audience design	1	1.962	.08	0.016
	ROS evaluation	1	5.404	.01	0.044
	ROS subjective comprehension	1	0.769	.19	0.007

^a^METI: Muenster Epistemic Trustworthiness Inventory.

^b^ROS: Recipient Orientation Scale.

## Discussion

### Investigation 1

In summary, the findings of the content analysis suggest that it might be more typical for the most relevant health videos on YouTube to use first-person pronouns compared with the most relevant health videos on Vimeo because the relative proportion of first-person pronouns used in each investigated video about *health* and *diet* was higher on YouTube than on Vimeo. On the other hand, there were no differences in the number of second-person pronouns. These findings supplement findings that suggest specific Web-based platforms their own typical language styles [68,69]. Although both platforms do not explicitly declare rules for how people should use language (despite vulgar language considering netiquette), people seem to use different words. Depending on implicit rules and norms of YouTube and Vimeo—which might result from the specific affordances of both platforms—users of both platforms might have evolved a typical communication behavior. According to LET and CAT, a specific language style might fit the purpose of presenting videos on a particular platform, address a specific audience more appropriately, or is perceived by other users as more appropriate. It would be of further interest to investigate not only whether users use different language styles on platforms beyond those investigated here (YouTube and Vimeo) but also whether they use different language styles in videos with different content, beyond those related to health and diet, which might represent a subcontext (or a subcommunity) of each Web-based platform. Moreover, the language styles might be a different change for videos that have comparatively lower rankings than the ones investigated here, which are more highly ranked. As highly ranked videos represent videos recommended by platforms (through opaque criteria), these videos are indeed likely to represent platforms’ *typical* videos but do not represent all available Web-based videos.

### Investigation 2

In summary, findings indicate differences in users’ general trust in investigated Web-based platforms. Participants seemed to trust in YouTube more than in Moodle to offer a Web-based video platform, as notably, participants’ familiarity and willingness to transact with YouTube were higher than with Moodle. In more detail, it is of special interest that participants’ trust-related evaluations in terms of the platform’s expertise, benevolence, and integrity did not differ for YouTube and Moodle although participants’ reported familiarity and willingness to transact differed for YouTube and Moodle. Accordingly, it seems worthwhile for further research to consider users’ trust attitudes and users’ trust actions toward Web-based platforms, as trust actions may not necessarily be caused by users’ actual trust attitudes. Instead, users may use a Web-based platform such as YouTube because it is popular although users may not necessarily believe this platform to be more competent, benevolent, or to have more integrity than other platforms [27,120].

### Investigation 3

#### Main Findings

Participants judged the credibility of information differently depending on whether the scientific explanations concluded that the associated nutrition myth was either confirmed or disconfirmed or neither confirmed nor disconfirmed by scientific evidence (H1). Interestingly, the credibility of information in videos was not judged depending on the language style of vloggers (H2) nor depending on the context of Web-based communication (H3). Hence, people seem not to consider the language style of vloggers or the context of communication when evaluating a video’s information. Although the language style of the vlogger impacted participants’ judgments of the vlogger’s benevolence (H2; ie, participants judged vloggers who used YouTube-typical language to be more benevolent than vloggers who used formal language), the video’s platform individually had no impact on participants’ judgments of the trustworthiness of the vlogger (H3). Although, contrary to the assumption that the appropriateness of a vlogger’s language style given a certain context of communication (ie, platform of video) should lead people to rate the vlogger’s credibility and trustworthiness higher, participants’ trust-related evaluations of the information and the providers were not affected by an interaction between the vlogger’s language style and the context of communication (H4). However, participants judged the vlogger’s YouTube-typical language style to be more accommodative (ie, appropriate, as adapted toward the addressed audience) regardless of the Web-based platform on which the video was presented. In addition, they ascribed more language accommodation in Moodle for the vlogger who used YouTube-typical language compared with formal language, whereas their ascription of language accommodation did not differ for formal and YouTube-typical language in YouTube. Thus, participants were reciprocally impacted by the context of Web-based communication and the provider’s language style, as they ascribed language appropriateness differently between YouTube and Moodle (H4).

To sum up, because participants did not judge the information or the vloggers on YouTube to be more reliable than on Moodle, the expected differences in participants’ trust in these platforms did not carry over into their judgments on reliability of the information and the vlogger (H3). Similarly, participants did not judge the information or the vloggers to be more reliable when those vloggers used formal versus YouTube-typical language (H2). Instead, participants judged vloggers to be more benevolent and their language use to be more accommodative toward the audience if the vloggers used YouTube-typical language, which might in turn indicate that people do not expect to identify whether a vlogger is or is not a competent expert according to their use of formal language alone. Perhaps, people perceive the use of YouTube-typical language in Web-based videos as being a common choice for Web-based videos in general. That would also explain why participants did not judge information or vloggers to be more reliable if the video contained a rather appropriate (ie, YouTube-typical language on YouTube and formal language on Moodle) compared with a rather inappropriate language style (ie, YouTube-typical language on Moodle and formal language on YouTube) but instead ascribed more accommodation for the vloggers who used YouTube-typical language compared with formal language on Moodle. At the same time, because participants did not ascribe the accommodation of the vlogger’s YouTube-typical and formal language use differently on YouTube, the use of YouTube-typical language is, perhaps, universal for Web-based videos. Together, these findings indicate rather complex interdependencies for how a vlogger’s language use and its *appropriateness* given a certain Web-based context affects people’s trust-related evaluations.

Finally, participants judged unclear explanations containing scientific evidence neither or nor against the nutrition myths (*coffee and dementia* and *too many diet beverages leading to diabetes*) to be less credible than those explanations concluding that the scientific evidence speaks either against (*cola and pretzel sticks* and *harmfulness of too many eggs*) or for (*healthy nutrition and cancer* and *the healthiness of low-salt diet*) the myth; this partially confirms the derived hypothesis (H1). Surprisingly, participants judged the credibility of explanations similarly when the explanations confirmed a myth and when they disconfirmed a myth. As people heuristically tend to make associations between how much effort they believe they put into understanding the given information and how they evaluate the information’s credibility [30,32], participants might have perceived that both forms of explanations that gave clear scientific evidence (either for or against the common myth) were credible because participants did not have to put much effort into processing the information. Although it is likely that ambiguous information is rather complex and that people, therefore, may use more effort to process this information, it is also interesting to reflect on potential differences for how confirming and disconfirming information is assessed.

#### Limitations and Implications

In addition to the findings summarized above, in the following, the limitations of this study’s third investigation will be emphasized. Furthermore, by taking into account the related research on health information seekers’ trust-related evaluations, the results and limitations of this study’s third investigation still emphasize the need for considering a complex interplay between single factors (ie, language style, information content, and the context of Web-based communication) when investigating seekers’ tendencies to rely on Web-based information and providers.

Against the assumption that different Web-based contexts would influence people’s judgments on the credibility of information and the trustworthiness of vloggers [34,70], videos were not judged differently depending on whether they were presented on YouTube or Moodle. In retrospect, there are factors lying in the manipulation of the experiment that might have influenced these findings. As videos on both platforms needed to be introduced by a lecturer to guarantee the valid and plausible comparison of a Web-based video platform and an academic Web-based platform, participants might have evaluated the video’s reliability by also considering the lecturer as a gatekeeper. Thereby, the presence of the lecturer might have caused participants to perceive the content as filtered through a professional gatekeeper with high expertise [30]. Therefore, potential differences between Moodle and YouTube related to the vlogger’s trustworthiness may have remained undetected because the gatekeeper may have caused the ratings to be relatively high to start out with (ceiling effect).

Furthermore, static screenshots of both platforms might have weakened the external validity, as participants were not able to interact with the platform’s usual features. As such, research that assumes a platform’s norms and rules will have an impact on people’s trust-related evaluations of the platform’s content might also need to consider both limitations of this study. Moreover, although the investigated platforms are limited to their specific features, which may represent plausible differences in platforms’ communities and people’s trust in Moodle and YouTube, people, in particular, may also differ in their frequency of using both platforms. Accordingly, the manipulation check showed that fewer participants remembered the screenshots of Moodle than of YouTube. This, in turn, may indicate differences in people’s information processing between both platforms; it is unclear whether participants may have unconsciously recognized the screenshots. However, further research might also focus on platforms that users are equally familiar with, so that researchers can control for potential differences in information processing when investigating whether the Web-based platforms influence how people evaluate the reliability of a platform’s content.

According to users’ trust in both platforms, another limitation lies in the comparison between participants of this study’s second and third investigations and, therefore, in the causal explanation for the derived third hypothesis. Although plausible, it is still unclear whether participants in the third investigation would have rated their trust in Moodle and YouTube similar to those participants in the second investigation. As participants in the third investigation had to be unaware of this study’s aim (ie, to investigate a potential impact of 2 Web-based platforms that would cause differences in participants’ reliability judgments), they had to be recruited independently from those recruited in the second investigation. Hence, even if it is likely that participants in the second investigation were equally representative as those in the third investigation, it is presumable that participants in the third investigation were at least more conversant with the specific type of Moodle called learnweb. As participants in the third investigation were recruited using a mailing list from the same university that provides this specific form of Moodle, the presentation of a screenshot showing this specific Moodle surface may have triggered a higher perceived familiarity with (and a higher trust in) Moodle compared with an introduction and written explanation about Moodle’s purpose and usage (presented within the second investigation). Accordingly, further research that is still aims to investigate if the context of Web-based communication affects information seekers’ trust-related evaluations might not only focus on different platforms with specific rules and norms but should also consider people’s experiences and habits in using these platforms. In this vein, different contexts of Web-based communication might not only be determined by different Web-based platforms but also within the same Web-based platform, as, for instance, subgenres within YouTube might evolve specific norms or rules [35].

In line with research on the credibility of information, participants in this investigation assessed the credibility of information depending on its uncertainty. In fact, it is interesting that the content was judged depending on whether it provided a strong conclusion about a common nutrition myth or whether it prevented a strong conclusion about the myth. Accordingly, the uncertainty of information may not only concern scientifically controversial and ambiguous findings but it may also entail the conflict between people’s conceptions of well-known (ie, allegedly to be true) information and scientific evidence for or against this information. Thereby, giving strong evidence either for or against a nutrition myth was judged as being more credible than giving ambiguous scientific findings that are unable to confirm or disconfirm the myth. Although research about the uncertainty of information indicates consistent information to be more credible than conflicting information, because of people’s tendency to evaluate the conflicting information in a way that is beneficial to their existing knowledge [51,95,98,100], the conflicting information in this study may reflect conflicts with rather low relevance. Although it is likely that well-known nutrition myths lead people to believe in these myths because of their familiarity and wide-spread nature [105], they still represent information that is less relevant to people’s social identity, needs, or interests [7]. Hence, participants in this study may not have been affected in a biased way to integrate the conflicting information into their existing knowledge, as busting a common nutrition myth may not be particularly relevant to a participant’s identity. Hence, it seems fruitful for further investigations on people’s credibility judgments to focus not only on provided information that just disconfirms or confirms existing information but also on the comparison between information that conflicts with people’s beliefs in various ways (relevance, scientific evidence, myths, beliefs, etc). However, taking into account people’s existing (mis)conceptions could help educate people, as they may judge information as more credible when they are given clear evidence for or against their existing (mis)conceptions; in addition, this new clear information may be easier for them to integrate into their existing knowledge structure [105].

### Summarizing Discussion

#### Principal Findings

This study’s investigations aimed to supplement research on people’s evaluations of information and information providers on the Web. As it can be challenging for (lay)people to evaluate information found on the internet based on academic criteria that indicate whether information is correct, complete, and appropriate, it is important to investigate what cues people use instead of academic criteria to evaluate the reliability of information; this is important not only for understanding people’s trust-related judgments but also for understanding how people process cues and make decisions. In this context, the main purpose of this study was to investigate whether the context of Web-based communication has an impact on how people evaluate the credibility of information and the information provider’s trustworthiness.

In line with the idea that the context of Web-based communication—along with its rules and norms—determines users’ language use and health information seekers’ proper understanding of information [5,36,37], this study’s first 2 investigations aimed to characterize the Web-based context of communication more concretely (RQ1). Therefore, the relationship between individuals and their expectations of a platform’s rules and norms [61] was investigated by focusing on users’ typical language use on YouTube and Vimeo and on users’ trust in YouTube and Moodle. In line with previous research on different language styles for specific Web-based platforms [68,69], health videos on YouTube seemed to use more first-person pronouns than health videos on Vimeo. It appears plausible that affordances of Web-based platforms constitute users’ typical language use as well as their expectations about how others should use language within these Web-based platforms [64]. Furthermore, participants in the second investigation trusted more in YouTube than in Moodle. These differences between users’ trust in Web-based platforms may likewise cause differences in users’ trust-related evaluations for the very same Web-based information or provider given on different Web-based platforms [34,70]. Although it is challenging to identify implicit norms and rules of platforms because of a constantly changing Web-based platform environment, findings of the content analysis study and the survey data support the assumptions that users use different language styles on certain Web-based platforms, and they also have different levels of trust for different Web-based platforms.

According to LET and CAT, the context of Web-based communication not only determines users’ typical language use but it may also affect health information seekers’ proper understanding of information, as seekers may make judgments based on the interplay of the information content, the provider’s language style, and the context of Web-based communication. Building on the first 2 investigations, in a Web-based experiment, we investigated whether health information seekers’ trust-related evaluations of a Web-based video about nutrition myths were influenced not only by the content of the information but also by the providers’ language style and the context of Web-based communication, where the context was the Web-based platform, either Moodle or YouTube (RQ2). Moreover, as research indicates that people’s willingness to rely on information and information providers on the Web is influenced in a complex manner [30,33], we experimentally investigated any individual and reciprocal impact of the following factors: the uncertainty of the information being presented in the video, the language use of the vlogger presenting the information, and the Web-based context of communication. Hence, specific aspects of the message, the source, and the media were investigated, as they are expected to influence not only the communication but also the evaluation of communication and, in turn, the communication success [36,37].

Accordingly, participants’ trust-related evaluations in the experiment study indicate rather complex interdependencies between health information seekers’ evaluations of the vlogger’s language style and the Web-based video platform. Hence, both the language style of the provider and the context of communication seemed to have an individual and reciprocal impact on seekers’ trust-related evaluations, but not in the expected manner. That is, although participants judged the credibility of information in videos equally regardless of the platform and the vlogger’s language use, for the videos presented on Moodle, participants thought that vloggers who used a YouTube-typical language were more accommodating toward the audience than vloggers who used formal language. Moreover, when participants assessed the trustworthiness of the vlogger, their judgments of her benevolence were affected by her language style; conversely, when participants assessed the credibility of information, their judgments were influenced by the uncertainty of information.

#### Limitations and Implications

Of note, all investigations in this study only emphasize the topic of health and nutrition. It is important to understand how people judge nutrition information found on the Web, as using the internet to seek out information about nutrition continues to increase [2,3], and people’s decisions based on this information may influence people’s health [6]. Future research could also focus on health information beyond explanations about common nutrition myths, as information seekers’ decisions about whom and which information to rely on could be biased in many ways. In this vein, a strong bias might result if the Web-based information conflicts with seekers’ motivational and emotional constitutions, such as might be the case when smokers hear health information about smoking [7]. Accordingly, information about nutrition myths might be less prone to be processed in a biased manner, as it could be less conflicting to seekers’ own attitudes. As we found that people evaluate the credibility of nutrition information differently based on how uncertain it is, namely that they find nutrition information more credible when the provided scientific evidence speaks either strongly for or against the common nutrition myth rather than when the evidence is inconclusive, providing nutrition information on the Web should take into account people’s existing knowledge about this information. Similarly, further research might investigate whether additional information might help people to more accurately judge provided information that relies on uncertain scientific evidence.

Considering the integrative investigation of factors, including the language style of the provider, the content of information, and the context of Web-based communication, all of this study’s investigations should be treated as an approach to conceptualize the context of Web-based communication and determine its importance for seeking health information on the Web. In line with communication theories [36,37], communication and its success are influenced by the context in which it takes place. In addition, especially health information seekers risk to misinterpret the correctness of the information, if they neglect the context of Web-based communication [5]. However, conceptualizing and operationalizing the context of Web-based communication have many challenges [56], and by considering the accompanying rules and norms of Web-based platforms that are constituted by a platform’s affordances and users’ use of these affordances [61], the way context is operationalized in this study fails to address other aspects that might be considered context [57]. Similarly, research to date has identified that there are several aspects important for seeking health information on the Web, such as seekers’ (epistemic) beliefs [30,95]. Thus, more aspects should be considered in future research in addition to the aspects investigated in this study. Furthermore, the language style of providers and the context of Web-based communication in this study were operationalized by specific aspects that do not capture all relevant aspects of language styles (eg, the use of technical jargon) and contexts of Web-based communication (eg, subgenres on YouTube). Furthermore, the Web-based environment is constantly evolving over time. Hence, seeking health information on the Web is also continually changing. In the future, seeking information on the Web might be complemented by completely new affordances of online media (eg, further developments of augmented reality). Hence, a generalization of the concrete context of Web-based communication investigated in this study is limited to some extent. Although defining context does somewhat limit the external validity, it does, however, take into account what might be the most common aspects of evaluating Web-based health information within specific Web-based environments. In this vein, this approach might capture health information seekers’ actual experiences in an even more valid way. It seems fruitful to consider additional Web-based platforms with different aspects that may constitute differences between users’ typical use of Web-based platforms when investigating Web-based health information seekers’ trust-related evaluations. As the definition of context refers to norms that are determined by affordances of online media and that entail users’ expectations about how to use cues for the specific media, further research might particularly highlight users’ expectations about (other) users’ media habits. In this vein, research on expectations will face some challenges (eg, pre-expectations have most often been induced instead of assessed directly, as this would prime their actual expectancies [121]). Accordingly, it might be useful for research to focus on users who are familiar with online media, such as frequent users. Unfortunately, the participants in this study’s experiment were rather infrequent users, and YouTube and Moodle were not used with the same frequency by participants. Hence, further research could investigate frequent users of Web-based platforms to identify if user expectations regarding the use of media affordances affect how users evaluate information and providers. In this context, it also seems valuable to explicitly ask people what they expect when using a typical platform.

#### Conclusions

Often, research on how health information seekers evaluate information providers’ trustworthiness and information credibility on the Web focuses on single aspects of either the provider or the information. Much less research has addressed how these factors are affected by the context of communication. By focusing on the Web-based platform as an entity that enables one to conceptualize and operationalize the context of Web-based communication, it seems fruitful to investigate the impact of this communication context on people’s reliability judgments, as the context of Web-based communication is expected not only to constitute the communication itself but also to influence the evaluation of communication and its communicative success. A future challenge, then, will be not only to specify the context of Web-based communication by identifying people’s expectations and uses derived from a platform’s affordances but also to investigate in a valid way the individual and reciprocal impact of individual Web-based factors on people’s willingness to rely on information and information providers. As the way people process Web-based information seems to be influenced in a complex manner, understanding how people rely on nutrition information has to consider various Web-based aspects and whether they have any individual and integrative impact on people’s evaluations.

## Appendix

Multimedia Appendix 1 Description of videos for YouTube and Vimeo video content analysis.

Multimedia Appendix 2 Introduction to inform about where the videos came from according to the experimental conditions.

Multimedia Appendix 3 Screenshots of experimental videos being embedded within a screenshot that showed the interface of either Moodle or YouTube.

Multimedia Appendix 4 Correlations for the dependent and control variables (experiment).

## Abbreviations

CAT: communication accommodation theory
LET: language expectancy theory
LIWC: Linguistic Inquiry and Word Count
METI: Muenster Epistemic Trustworthiness Inventory
ROS: Recipient Orientation Scale
RQ: research question
