# Supplementary material for: Investigating the Role of Communication for Information Seekers’ Trust-Related Evaluations of Health Videos on the Web: Content Analysis, Survey Data, and Experiment
Source: Interact J Med Res. 2018 Dec 21;7(2):e10282. doi: 10.2196/10282 (PMC6322917; doi:10.2196/10282)
Supplement: Multimedia Appendix 1 [file ijmr_v7i2e10282_app1.pdf]

Description of Videos for Content Analysis

Table 1

Descriptive values of video search

platform	Search term	N (%)	sex of voice
YouTube	“health”	11	18 female/7 male
	“diet”	14	
	Total	25	
Vimeo	“health”	9	2 female/9 male
	“diet”	2	
	Total	11	
	Total	36	21 female /16 male

Note. Overall 200 videos (50 per search term) were judged whether they are going to be analyzed or excluded because of previously determined criteria.

Table 2

Overview of all transcribed videos

Platform	Search Term	Original rank of video	Presence of a protagonist	Duration time	Declared/apparent topic/aim of video	Video (Titel, Link)
Vimeo	diet	17.	Yes	4.52	"Diet: The Disease of our Generation"	https://vimeo.com/112832255
		22.	Yes	6.49	"The Fat Diet"	https://vimeo.com/72150860
	health	4.	No	1.03	„Men's Health // How a Bean Becomes a Fart"	https://vimeo.com/103721959
		5.	No	1.56	„ CRC Health Group: Health & Welfare"	https://vimeo.com/32216194
		6.	No	0.30	„ Dignity Health "	https://vimeo.com/155463374
		24.	No	1.20	„ Beer Goggles Explained - Men's Health "	https://vimeo.com/91322930
		25.	Yes	3.27	„ Sugar - Health Nuts"	https://vimeo.com/44273084
		29.	No	1.41	"Total Health Care | Affordable Care Act FAQ"	https://vimeo.com/92149026
		36.	No	1.19	„ How High BP Causes a Stroke / Men's Health Magazine "	https://vimeo.com/97006830
		48.	Yes	4.06	„ Rastovich's Yoga Routine - Health Nuts"	https://vimeo.com/14492968
		49.	Yes	3.25	"Sunburn Basics - Health Nuts"	https://vimeo.com/41601463
YouTube	diet	5.	Yes	3.12	"Easy Diet Hack + My #1 Healthy Snack Idea ♥ Easy Diet Tips ♥ Wengie ♥"	https://www.youtube.com/watch?v=owMzIgGpk94
		6.	Yes	9.37	„10 CRAZIEST Diet Trends EVER"	https://www.youtube.com/watch?v=S27THWdOxss
		7.	Yes	3.01	„What I Eat In a Day: Elimination Diet"	https://www.youtube.com/watch?v=acVD0n7Sd80
		8.	No	2.27	„Exercise vs Diet"	https://www.youtube.com/watch?v=ztiHRiFXtoc
		9.	Yes	9.01	„3 day Military Diet"	https://www.youtube.com/watch?v=YQhoQUxap6o
		10.	Yes	7.13	„HOW TO GET HEALTHY! My Diet Tips"	https://www.youtube.com/watch?v=VJRsjUY4PGI
		12.	Yes	7.57	"HOW TO LOSE 10 POUNDS IN 3 DAYS | Military Diet, Does It Really Work? *NEW*"	https://www.youtube.com/watch?v=L4Ev17To2XY
		14.	No	3.25	„HOW TO LOSE WEIGHT FAST 10Kg in 10 Days"	https://www.youtube.com/watch?v=aGtwMA5_mUo
		15.	Yes	6.19	„Diet to Lose Weight | What I Eat In A Day | Burn Fats Diet"	https://www.youtube.com/watch?v=AiCnERkm2Jw
		24.	Yes	9.10	„How I Lost 10 Pounds + Diet Tips"	https://www.youtube.com/watch?v=Ci0gjKhImCo
		31.	Yes	4.03	"The Military Diet: How To Lose 10 Pounds in 3 Days"	https://www.youtube.com/watch?v=Ht-UV3nutgM
		36.	Yes	8.44	„My Fitness Routine | Diet, Exercise & More!"	https://www.youtube.com/watch?v=bh9XVnSlUpY
		46.	Yes	7.43	„THE MILITARY DIET: does it really work? find out how much weight I lost!"	https://www.youtube.com/watch?v=eUpG91E50_k
		49.	Yes	4.56	"My Healthy Diet Routine: Get Slim For Summer! + School Lunch & Snack Ideas!"	https://www.youtube.com/watch?v=c5vEbjBXHwg
	health	3.	Yes	4.36	„Tips for a healthy lifestyle"	https://www.youtube.com/watch?v=0aNNYEUARAk
		4.	Yes	3.48	„ What Your Lip Color Says About	https://www.youtube.com/watch?

			Your Health “	v=_BKPqICyn9s
5.	Yes	6.56	„ 10 Health Myths People Still Believe”	https://www.youtube.com/watch?v=-rISIRTYmQ8
6.	Yes	5.05	„ Health & Fitness Life Hacks!”	https://www.youtube.com/watch?v=VNy31THi7i8
9.	Yes	4.19	„ What is Exposure Therapy? PTSD, Anxiety, OCD and more... Mental Health Help with Kati Morton”	https://www.youtube.com/watch?v=mlJy85EQrCI
15.	No	4.01	“Women's Pelvic Health: 1 Female Reproductive Anatomy”	https://www.youtube.com/watch?v=4P4nMQixnHY
19.	Yes	8.42	“Fitness & Health Motivation, Life Hacks + DIY Inspiration Board”	https://www.youtube.com/watch?v=zES85siew4c
27.	Yes	3.40	„Health News in Brief 25 JUL 2016 (Beta)“	https://www.youtube.com/watch?v=HQIFoeA_ga0
31.	Yes	4.03	„ 5 Things Your Nails Can Say About Your Health“	https://www.youtube.com/watch?v=aTSVHwzkYI4
34.	No	5.10	„How do carbohydrates impact your health? - Richard J. Wood”	https://www.youtube.com/watch?v=wxzc_2c6GMg
45.	No	3.24	“Men health animation”	https://www.youtube.com/watch?v=LgmZsitCS1U
