# Supplementary material for: Investigating the Role of Communication for Information Seekers’ Trust-Related Evaluations of Health Videos on the Web: Content Analysis, Survey Data, and Experiment
Source: Interact J Med Res. 2018 Dec 21;7(2):e10282. doi: 10.2196/10282 (PMC6322917; doi:10.2196/10282)
Supplement: Multimedia Appendix 2 [file ijmr_v7i2e10282_app2.pdf]

Introduction to inform about where the videos came from according to the experimental conditions

Table 1

Introduction to inform about where the videos came from according to the experimental conditions.

Between - Instruction - “YouTube”	Between - Instruction - “Moodle”
Information about the selected video<br><br>Dr. Windscheit, nutritional science lecturer, gives an undergraduate class on principles of nutritional science. This course takes place for 2 hours every week. In addition, the classes, lecturer Dr. Windscheit occasionally sends the students additional material by mail. Here you see a mail in which he refers to a YouTube video.<br>In the following you will see this video and you will be asked questions about the video as well as the person giving information in the video.<br><br>“Dear students,<br><br>For your information, I came across a video on YouTube. The video deals with typical nutritional myths and their scientific background. Please take a look at the video before our next class. This is the direct link to the video on YouTube:<br>https://www.youtube.com/watch?v=yOUq6IFfbTY<br><br>Kind regards,<br>F. Windscheit”	Information about the selected video<br><br>Dr. Windscheit, nutritional science lecturer, gives an undergraduate class on principles of nutritional science. This course in the extend of 2 hours takes place weekly. In addition, the classes, lecturer Dr. Windscheit occasionally sends the students additional material by mail. Here you see a mail in which he refers to a video to be found on the university’s learning platform. In the following you will see this video and you will be asked questions about the video as well as the person giving information in the video.<br><br>“Dear students,<br><br>For your information, I have uploaded a video on Learnweb. The video deals with typical nutritional myths and their scientific background. Please take a look at the video before our next class. This is the direct link to Learnweb<br>https://www.uni-muenster.de/LearnWeb/learnweb2/mod/resource/view.php?id=837734<br><br>Kind regards,<br>F. Windscheit”

Note: “Learnweb” is the specific name of Moodle which is used by the university from which the participants in the experiment came from.
