# Supplementary material for: Investigating the Role of Communication for Information Seekers’ Trust-Related Evaluations of Health Videos on the Web: Content Analysis, Survey Data, and Experiment
Source: Interact J Med Res. 2018 Dec 21;7(2):e10282. doi: 10.2196/10282 (PMC6322917; doi:10.2196/10282)
Supplement: Multimedia Appendix 3 [file ijmr_v7i2e10282_app3.pdf]

Screenshots of experimental videos being embedded within a screenshot that showed the interface of either Moodle or YouTube

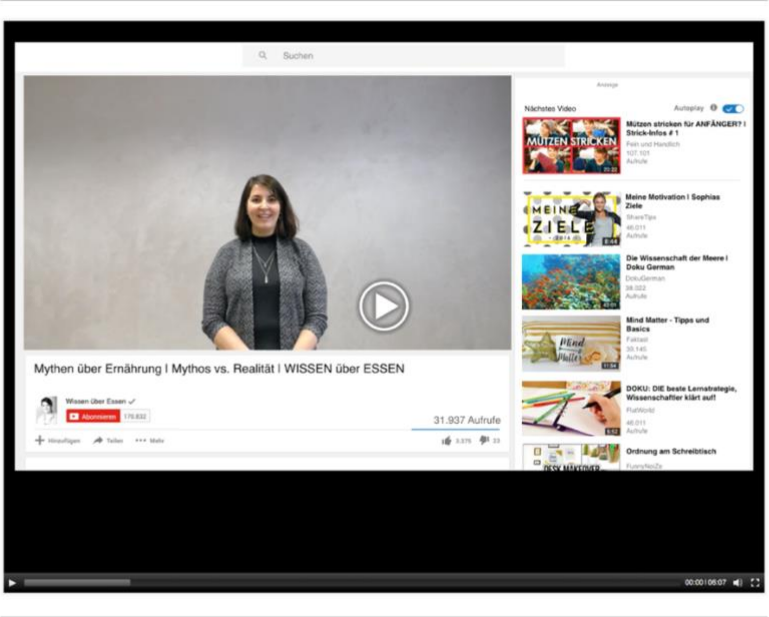

Figure 1. Video about nutrition myths being embedded within a screenshot that showed YouTube.

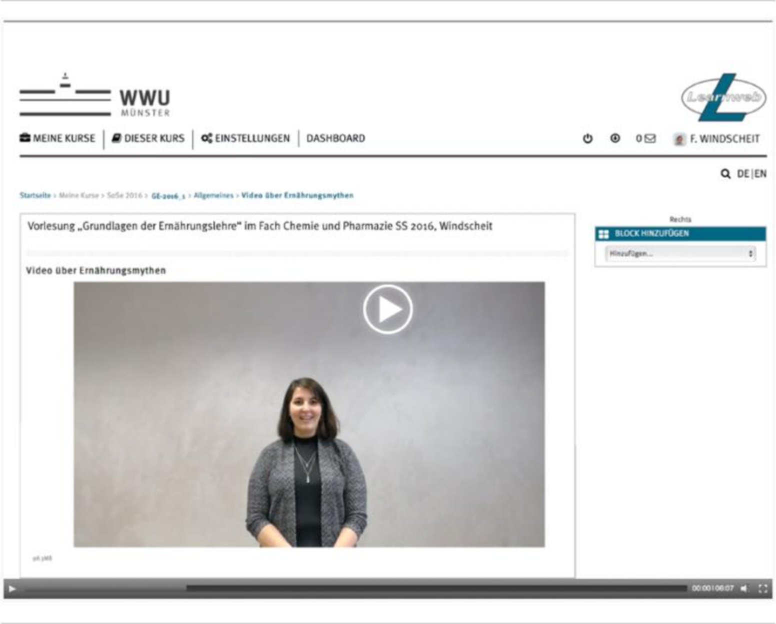

Figure 2. Video about nutrition myths being embedded within a screenshot that showed Moodle.

For videos and transcribed texts, please refer to
Https://drive.google.com/drive/folders/1j8poFJ3swpaZdRZOXGt7njmry3NfZ7FC?usp=sharing
