# Supplementary material for: Investigating the Role of Communication for Information Seekers’ Trust-Related Evaluations of Health Videos on the Web: Content Analysis, Survey Data, and Experiment
Source: Interact J Med Res. 2018 Dec 21;7(2):e10282. doi: 10.2196/10282 (PMC6322917; doi:10.2196/10282)
Supplement: Multimedia Appendix 4 [file ijmr_v7i2e10282_app4.pdf]

Correlations for the Dependent and Control variables (experiment).

Table 1

Correlations for dependent and control variables for the third investigation (experiment).

Correlations

		sex	age	Frequency of computer use	Frequency of Internet use	Frequency of watching online videos (3 Items)	Preknowledge on nutrition (4 Items)	Relevance of video	METISCORE	METIcompetence	METintegrity	METI benevolence	ROS SCORE	ROS audiencedesign
sex	Pearson corr.	1	,129	,293**	,120	-,209*	-,102	,219*	,135	,140	,130	,069	-,118	-,123
	Sign. (2-sided)		,154	,001	,187	,020	,257	,015	,136	,121	,150	,445	,191	,172
	N	124	124	124	123	124	124	124	124	124	124	124	124	124
age	Pearson corr.	,129	1	,095	-,111	,206*	-,031	-,065	,184*	,219*	,143	,079	-,200*	-,163
	Sign. (2-sided)	,154		,295	,220	,021	,730	,471	,041	,014	,114	,386	,026	,071
	N	124	124	124	123	124	124	124	124	124	124	124	124	124
Frequency of computer use	Pearson corr.	,293**	,095	1	,537**	-,116	,054	,081	,019	,025	,018	,001	-,062	-,069
	Sign. (2-sided)	,001	,295		,000	,199	,554	,369	,833	,780	,839	,994	,492	,447
	N	124	124	124	123	124	124	124	124	124	124	124	124	124
Frequency of Internet use	Pearson corr.	,120	-,111	,537**	1	-,094	-,074	-,143	-,064	-,067	-,025	-,066	-,037	-,035
	Sign. (2-sided)	,187	,220	,000		,300	,414	,114	,484	,462	,782	,469	,687	,702
	N	123	123	123	123	123	123	123	123	123	123	123	123	123
Frequency of watching online videos	Pearson corr.	-,209*	,206*	-,116	-,094	1	-,003	-,186*	,008	,084	-,016	-,101	,029	,071
	Sign. (2-sided)	,020	,021	,199	,300		,969	,039	,927	,354	,861	,263	,748	,432
	N	124	124	124	123	124	124	124	124	124	124	124	124	124
Preknowledge on nutrition	Pearson corr.	-,102	-,031	,054	-,074	-,003	1	,020	-,049	-,029	-,097	-,017	,092	,040
	Sign. (2-sided)	,257	,730	,554	,414	,969		,823	,585	,749	,284	,848	,307	,661
	N	124	124	124	123	124	124	124	124	124	124	124	124	124
Relevance of video	Pearson corr.	,219*	-,065	,081	-,143	-,186*	,020	1	-,463**	-,465**	-,413**	-,299**	,412**	,300**
	Sign. (2-sided)	,015	,471	,369	,114	,039	,823		,000	,000	,000	,001	,000	,001
	N	124	124	124	123	124	124	124	124	124	124	124	124	124
METI SCORE	Pearson corr.	,135	,184*	,019	-,064	,008	-,049	-,463**	1	,898**	,897**	,820**	-,534**	-,441**
	Sign. (2-sided)	,136	,041	,833	,484	,927	,585	,000		,000	,000	,000	,000	,000
	N	124	124	124	123	124	124	124	124	124	124	124	124	124
METI competence	Pearson corr.	,140	,219*	,025	-,067	,084	-,029	-,465**	,898**	1	,678**	,525**	-,490**	-,344**
	Sign. (2-sided)	,121	,014	,780	,462	,354	,749	,000	,000		,000	,000	,000	,000
	N	124	124	124	123	124	124	124	124	124	124	124	124	124
METI integrity	Pearson corr.	,130	,143	,018	-,025	-,016	-,097	-,413**	,897**	,678**	1	,770**	-,493**	-,442**
	Sign. (2-sided)	,150	,114	,839	,782	,861	,284	,000	,000	,000		,000	,000	,000
	N	124	124	124	123	124	124	124	124	124	124	124	124	124
METI	Pearson corr.	,069	,079	,001	-,066	-,101	-,017	-,299**	,820**	,525**	,770**	1	-,407**	-,408**
	Sign. (2-sided)	,445	,386	,994	,469	,263	,848	,001	,000	,000	,000		,000	,000

benevolence	N	124	124	124	123	124	124	124	124	124	124	124	124	124
ROS SCORE	Pearson corr.	-,118	-,200*	-,062	-,037	,029	,092	,412**	-,534**	-,490**	-,493**	-,407**	1	,933**
	Sign. (2-sided)	,191	,026	,492	,687	,748	,307	,000	,000	,000	,000	,000		,000
	N	124	124	124	123	124	124	124	124	124	124	124	124	124
ROS audience design	Pearson corr.	-,123	-,163	-,069	-,035	,071	,040	,300**	-,441**	-,344**	-,442**	-,408**	,933**	1
	Sign. (2-sided)	,172	,071	,447	,702	,432	,661	,001	,000	,000	,000	,000	,000	
	N	124	124	124	123	124	124	124	124	124	124	124	124	124
ROS evaluation	Pearson corr.	-,063	-,231**	-,006	,002	-,117	,108	,460**	-,559**	-,642**	-,446**	-,271**	,771**	,536**
	Sign. (2-sided)	,486	,010	,943	,984	,195	,234	,000	,000	,000	,000	,002	,000	,000
	N	124	124	124	123	124	124	124	124	124	124	124	124	124
ROS subj comprehension	Pearson corr.	-,073	-,017	-,083	-,082	,160	,135	,197*	-,153	-,080	-,168	-,194*	,498**	,392**
	Sign. (2-sided)	,423	,848	,362	,367	,076	,136	,028	,089	,374	,062	,031	,000	,000
	N	124	124	124	123	124	124	124	124	124	124	124	124	124
Coffee	Pearson corr.	,158	,071	,098	,028	-,165	,079	,533**	-,432**	-,438**	-,353**	-,303**	,461**	,366**
credibility	Sign. (2-sided)	,079	,434	,276	,756	,067	,381	,000	,000	,000	,000	,001	,000	,000
	N	124	124	124	123	124	124	124	124	124	124	124	124	124
Cola	Pearson corr.	,036	,111	,130	-,003	-,024	,088	,517**	-,431**	-,433**	-,357**	-,303**	,303**	,206*
credibility	Sign. (2-sided)	,695	,219	,150	,972	,791	,330	,000	,000	,000	,000	,001	,001	,022
	N	124	124	124	123	124	124	124	124	124	124	124	124	124
Light	Pearson corr.	,093	,144	,149	,026	-,030	,125	,442**	-,345**	-,366**	-,300**	-,197*	,439**	,354**
credibility	Sign. (2-sided)	,306	,111	,098	,779	,743	,168	,000	,000	,000	,001	,029	,000	,000
	N	124	124	124	123	124	124	124	124	124	124	124	124	124
Cancer	Pearson corr.	,142	,099	,052	,000	-,011	,052	,515**	-,403**	-,422**	-,327**	-,262**	,301**	,212*
credibility	Sign. (2-sided)	,115	,273	,563	,996	,900	,563	,000	,000	,000	,000	,003	,001	,018
	N	124	124	124	123	124	124	124	124	124	124	124	124	124
Salt credibility	Pearson corr.	,054	-,016	,031	,031	-,074	,147	,467**	-,455**	-,509**	-,373**	-,234**	,344**	,246**
	Sign. (2-sided)	,554	,863	,729	,731	,416	,104	,000	,000	,000	,000	,009	,000	,006
	N	124	124	124	123	124	124	124	124	124	124	124	124	124
Eggs credibility	Pearson corr.	,158	,056	,084	,083	-,048	-,077	,449**	-,566**	-,526**	-,485**	-,456**	,406**	,313**
	Sign. (2-sided)	,079	,537	,352	,362	,596	,395	,000	,000	,000	,000	,000	,000	,000
	N	124	124	124	123	124	124	124	124	124	124	124	124	124

Correlations

		ROS evaluation	ROS subj comprehension	Coffee & Dementia credibility	Cola credibility	Light Beverages credibility	Cancer credibility	Salt credibility	Eggs credibility
Geschlecht	Pearson corr.	-,063	-,073	,158	,036	,093	,142	,054	,158
	Sign. (2-sided)	,486	,423	,079	,695	,306	,115	,554	,079
	N	124	124	124	124	124	124	124	124
Alter	Pearson corr.	-,231**	-,017	,071	,111	,144	,099	-,016	,056
	Sign. (2-sided)	,010	,848	,434	,219	,111	,273	,863	,537
	N	124	124	124	124	124	124	124	124
Computernutzung	Pearson corr.	-,006	-,083	,098	,130	,149	,052	,031	,084

	Sign. (2-sided)	,943	,362	,276	,150	,098	,563	,729	,352
	N	124	124	124	124	124	124	124	124
Internetnutzung	Pearson corr.	,002	-,082	,028	-,003	,026	,000	,031	,083
	Sign. (2-sided)	,984	,367	,756	,972	,779	,996	,731	,362
	N	123	123	123	123	123	123	123	123
Online_gesamtnutzung	Pearson corr.	-,117	,160	-,165	-,024	-,030	-,011	-,074	-,048
	Sign. (2-sided)	,195	,076	,067	,791	,743	,900	,416	,596
	N	124	124	124	124	124	124	124	124
ernaehrung_beschaeftigung	Pearson corr.	,108	,135	,079	,088	,125	,052	,147	-,077
	Sign. (2-sided)	,234	,136	,381	,330	,168	,563	,104	,395
	N	124	124	124	124	124	124	124	124
videorelevanz_gesamt	Pearson corr.	,460**	,197*	,533**	,517**	,442**	,515**	,467**	,449**
	Sign. (2-sided)	,000	,028	,000	,000	,000	,000	,000	,000
	N	124	124	124	124	124	124	124	124
METI SCORE	Pearson corr.	-,559**	-,153	-,432**	-,431**	-,345**	-,403**	-,455**	-,566**
	Sign. (2-sided)	,000	,089	,000	,000	,000	,000	,000	,000
	N	124	124	124	124	124	124	124	124
	Pearson corr.	-,642**	-,080	-,438**	-,433**	-,366**	-,422**	-,509**	-,526**
	Sign. (2-sided)	,000	,374	,000	,000	,000	,000	,000	,000
METI competence	N	124	124	124	124	124	124	124	124
METI integrity	Pearson corr.	-,446**	-,168	-,353**	-,357**	-,300**	-,327**	-,373**	-,485**
	Sign. (2-sided)	,000	,062	,000	,000	,001	,000	,000	,000
	N	124	124	124	124	124	124	124	124
METI benevolence	Pearson corr.	-,271**	-,194*	-,303**	-,303**	-,197*	-,262**	-,234**	-,456**
	Sign. (2-sided)	,002	,031	,001	,001	,029	,003	,009	,000
	N	124	124	124	124	124	124	124	124
ROS SCORE	Pearson corr.	,771**	,498**	,461**	,303**	,439**	,301**	,344**	,406**
	Sign. (2-sided)	,000	,000	,000	,001	,000	,001	,000	,000
	N	124	124	124	124	124	124	124	124
ROS audience design	Pearson corr.	,536**	,392**	,366**	,206*	,354**	,212*	,246**	,313**
	Sign. (2-sided)	,000	,000	,000	,022	,000	,018	,006	,000
	N	124	124	124	124	124	124	124	124
ROS evaluation	Pearson corr.	1	,160	,444**	,349**	,427**	,346**	,399**	,448**
	Sign. (2-sided)		,076	,000	,000	,000	,000	,000	,000
	N	124	124	124	124	124	124	124	124
ROS subj comprehension	Pearson corr.	,160	1	,266**	,178*	,225*	,148	,146	,142
	Sign. (2-sided)	,076		,003	,048	,012	,102	,105	,115
	N	124	124	124	124	124	124	124	124
Coffee & Dementia	Pearson corr.	,444**	,266**	1	,636**	,702**	,644**	,568**	,601**
Credibility	Sign. (2-sided)	,000	,003		,000	,000	,000	,000	,000
	N	124	124	124	124	124	124	124	124
Cola credibility	Pearson corr.	,349**	,178*	,636**	1	,598**	,634**	,552**	,510**
	Sign. (2-sided)	,000	,048	,000		,000	,000	,000	,000

	N	124	124	124	124	124	124	124	124
Light Beverages credibility	Pearson corr.	,427**	,225*	,702**	,598**	1	,669**	,617**	,546**
	Sign. (2-sided)	,000	,012	,000	,000		,000	,000	,000
	N	124	124	124	124	124	124	124	124
cancer credibility	Pearson corr.	,346**	,148	,644**	,634**	,669**	1	,622**	,591**
	Sign. (2-sided)	,000	,102	,000	,000	,000		,000	,000
	N	124	124	124	124	124	124	124	124
Salt credibility	Pearson corr.	,399**	,146	,568**	,552**	,617**	,622**	1	,621**
	Sign. (2-sided)	,000	,105	,000	,000	,000	,000		,000
	N	124	124	124	124	124	124	124	124
Eggs credibility	Pearson corr.	,448**	,142	,601**	,510**	,546**	,591**	,621**	1
	Sign. (2-sided)	,000	,115	,000	,000	,000	,000	,000	
	N	124	124	124	124	124	124	124	124

**. The correlation is for an alpha-niveau of 0,01 (2-sided) significant.

*. The correlation is for an alpha-niveau of 0,05 (2-sided) significant.
